# Machine learning predictions of T cell antigen specificity from intracellular calcium dynamics

**DOI:** 10.1126/sciadv.adk2298

**Published:** 2024-03-06

**Authors:** Sébastien This, Santiago Costantino, Heather J. Melichar

**Affiliations:** ^1^ Centre de recherche de l'Hôpital Maisonneuve-Rosemont, Montréal, Québec, Canada.; ^2^ Département de Microbiologie, Infectiologie et Immunologie, Université de Montréal, Montréal, Québec, Canada.; ^3^ Department of Microbiology and Immunology, Goodman Cancer Institute, McGill University, Montréal, Québec, Canada.; ^4^ Département d’Ophtalmologie, Université de Montréal, Montréal, Québec, Canada.; ^5^ Département de Médecine, Université de Montréal, Montréal, Québec, Canada.

## Abstract

Adoptive T cell therapies rely on the production of T cells with an antigen receptor that directs their specificity toward tumor-specific antigens. Methods for identifying relevant T cell receptor (TCR) sequences, predominantly achieved through the enrichment of antigen-specific T cells, represent a major bottleneck in the production of TCR-engineered cell therapies. Fluctuation of intracellular calcium is a proximal readout of TCR signaling and candidate marker for antigen-specific T cell identification that does not require T cell expansion; however, calcium fluctuations downstream of TCR engagement are highly variable. We propose that machine learning algorithms may allow for T cell classification from complex datasets such as polyclonal T cell signaling events. Using deep learning tools, we demonstrate accurate prediction of TCR-transgenic CD8^+^ T cell activation based on calcium fluctuations and test the algorithm against T cells bearing a distinct TCR as well as polyclonal T cells. This provides the foundation for an antigen-specific TCR sequence identification pipeline for adoptive T cell therapies.

## Teaser

Artificial intelligence-assisted analysis of intracellular calcium dynamics allows for the rapid prediction of T cell specificity.

## INTRODUCTION

Adoptive T cell therapies are revolutionizing cancer treatment. In this context, T cells are engineered to redirect their specificity toward cancer antigens with either a chimeric antigen receptor or a predetermined, tumor-specific T cell receptor (TCR). TCR–T cell therapies have the potential to recognize antigens that arise from mutations, fusion proteins, and aberrantly expressed regions of the genome, thereby increasing the breadth of targets (*1*, *2*). However, the identification of tumor-specific TCRs is challenging due, in part, to the need to recognize patient-specific tumor antigens presented in the context of highly polymorphic major histocompatibility complex (MHC) molecules.

Despite recent advances in the field of computational biology, in silico prediction of antigen-specific TCR sequences is still ineffective (*3*). Current TCR identification platforms rely on in vitro selection of antigen-specific T cells for subsequent TCR sequencing. These techniques often depend on the ability of individual T cells to bind peptide-MHC (pMHC) multimers or their capacity to proliferate and/or express activation markers after in vitro peptide stimulation (*4*). As such, these methods may introduce biases toward selection of high-affinity TCR sequences, which undergo more robust pMHC binding and proliferation. In addition, recent work has shown that T cells bearing antigen receptors of low and high affinity for a given antigen perform different functions. While T cells bearing high affinity antigen receptors may, acutely, be more effective in their antitumor activity, they may also be more susceptible to inhibitory receptor mediated dysfunction and, potentially, off-target cross-reactivity (*2*, *5*–*12*). Thus, it may be important to consider engineering T cells with a breadth of TCR affinities for optimal therapeutic efficacy.

In this context, antigen-specific T cell identification based on calcium (Ca^2+^) oscillations downstream of TCR signaling is an alternative approach with notable potential. The kinetics of TCR-dependent increases in intracellular Ca^2+^ have been well described following in vitro and in vivo T cell activation (*13*). TCR engagement induces temporal oscillations in intracellular Ca^2+^ concentrations, with sustained, high intracellular Ca^2+^ levels associated with strong stimulation. The dynamics (amplitude, rate of oscillation, return to baseline, etc.) of intracellular Ca^2+^ fluctuations are dependent on the cellular system as well as the characteristics of the interaction between the T cells and the antigen-presenting cells (APCs) (duration of interaction, costimulation levels, cytokine milieu, etc.) (*14*–*18*). Furthermore, increases in intracellular Ca^2+^ concentrations are a proximal readout of TCR activation, occurring within seconds of antigen receptor stimulation, limiting the potential selection biases induced by prolonged interaction with an antigen and expansion of a potentially limited number of clones. Genetic reporters for Ca^2+^ signaling (e.g., nuclear factor of activated T cells–green fluorescent protein) have previously been used for the isolation antigen-specific TCR transduced T cells using a microfluidics system (*19*, *20*), but their use for the discovery of antigen-specific TCR sequences from polyclonal T cells has not yet been achieved. The complexity of TCR-dependent Ca^2+^ signals and the possibility that TCR-independent processes affect intracellular Ca^2+^ levels are hurdles for its widespread use as a marker for TCR activation.

The use of supervised machine learning (ML) tools to process highly complex phenomena is revolutionizing approaches to clinical and fundamental research (*21*–*24*). Several studies have shown that these methods can be used to characterize T cell antigen specificity from microscopy-based image datasets by monitoring the interaction of T cells with APCs or the autofluorescence changes that correlate with metabolic state (*25*–*27*). We propose to use ML algorithms, trained to identify TCR-dependent Ca^2+^ fluctuations, to provide a prediction of antigen specificity at the single-cell level.

Here, we present a proof-of-concept study for predicting T cell antigen specificity based on intracellular Ca^2+^ dynamics. We took advantage of TCR-transgenic T cells of known specificity, intracellular Ca^2+^ concentration indicator dyes, and simple imaging techniques to train and validate a ML model to accurately and efficiently predict antigen-specific T cells based on intracellular Ca^2+^ dynamics, which was then applied to polyclonal T cell responses. We show that convolutional neural networks (CNNs) allow for efficient and accurate prediction of T cell activation from intracellular Ca^2+^ fluctuations at early time points, matching or surpassing other ML approaches. This method also demonstrates the feasibility of training algorithms on monoclonal TCR-transgenic T cells, stimulated with model peptides, for the prediction of antigen specificity in polyclonal T cell responses.

## RESULTS

### In vitro T cell activation model to track intracellular Ca^2+^ dynamics

**Fig. 1.**
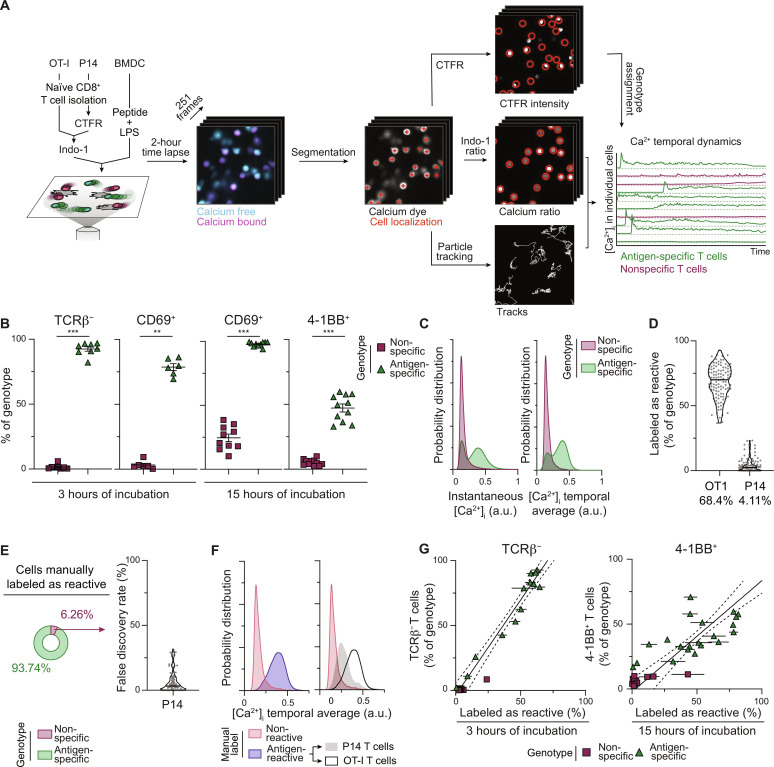
In vitro T cell activation model for the study of intracellular Ca^2+^ dynamics. (**A**) Schematic representation of the analysis pipeline. (**B**) Flow cytometry assessment of surface TCRβ down-regulation, CD69 expression, and 4-1BB expression, 3 or 15 hours after coculture with OVA peptide. Error bars indicate SD (*n* = 6 to 10 independent wells over four independent experiments; Mann-Whitney *U* test). (**C**) Distribution of intracellular Ca^2+^ concentration for all time points (left) or the average Ca^2+^ concentration over the entire time lapse (right) according to antigen specificity assignment (*n* = 7173 antigen-specific and *n* = 7564 nonspecific T cells over eight independent experiments). a.u., arbitrary units. (**D**) Frequency of T cells manually labeled as antigen-reactive. Horizontal lines show the median and numbers below show the average. Individual fields of view are represented in gray (*n* = 111 fields of view over eight independent experiments). (**E**) Proportion of antigen-specific and nonspecific cells among those manually labeled as antigen-reactive. (**F**) Distribution of the average intracellular Ca^2+^ concentration according to manual assignment and genotype (*n* = 5038 OT-I and *n* = 347 P14 cells labeled as antigen-reactive; *n* = 9351 cells labeled as nonreactive over eight independent experiments). (**G**) Correlation between the frequency of cells manually labeled as antigen-reactive and the frequency of cells down-regulating surface TCRβ or expressing 4-1BB, as measured by flow cytometry, after 3 or 15 hours of incubation. For each well, data for antigen-specific and nonspecific T cells are shown, and the frequency of manual labeling for all fields of view per well is averaged. Error bars indicate SEM, full line shows linear regression on antigen-specific T cells, and dotted lines show 95% confidence interval [TCRβ: *n* = 13 independent wells over four independent experiments, coefficient of determination (*R*^2^) = 0.9534, ρ = 0.9764; 4-1BB: *n* = 21 independent wells over six independent experiments; *R*^2^ = 0.442, ρ = 0.649]. OT-I, antigen-specific; P14, nonspecific. [Ca^2+^]_i_, intracellular calcium concentration. **P < 0.01 and ***P < 0.005.

For the purpose of training an ML algorithm that predicts T cell antigen specificity based on Ca^2+^ dynamics, we developed a simple imaging and analysis pipeline (Fig. 1A). To generate a widely applicable and more physiologically relevant in vitro system, we chose to develop an assay that uses peptide rather than pan–T cell stimulation (e.g., anti-CD3ε/CD28 antibodies or phytohaemagglutinin). With this method of stimulation, polyclonal T cells are poorly suited for training ML algorithms due to the very low frequency of antigen-specific T cells and the inability to know a priori the antigen reactivity of individual cells. Standard ML training requires labeled ground-truth data and balanced datasets, with a similar number of positive and negative cells. Therefore, we used murine monoclonal OT-I and P14 TCR-transgenic naïve CD8^+^ T cells in combination with lipopolysaccharide (LPS)–matured bone marrow–derived dendritic cells (BMDCs), loaded with chicken ovalbumin (OVA) 257-264 peptide (fig. S1). CD8^+^ T cells are labeled with a ratiometric Ca^2+^ indicator dye, Indo-1, where the ratio between Ca^2+^-free and Ca^2+^-bound emission wavelengths is indicative of relative intracellular Ca^2+^ concentration. Unless otherwise noted, both OT-I and P14 T cells are cocultured in the same well at a 1:1 ratio; a vital cytoplasmic stain, CellTrace Far Red (CTFR), was used to label either OT-I or P14 T cells before Indo-1 staining to differentiate the two populations. Images for both Indo-1 and CTFR were captured over a period of 2 hours, beginning a few minutes after the start of the coculture, to monitor intracellular Ca^2+^ dynamics. An in silico analysis pipeline was generated to automatically identify each cell, track it over time, measure the fluorescence of Indo-1 at each time point, and assign a genotype based on CTFR fluorescence. Thus, we can measure the dynamics of intracellular Ca^2+^ concentration for individual cells and know their antigen specificity.

For initial validation of the in vitro assay, we assessed T cell activation using well-established flow cytometric analysis of early (TCRβ down-regulation and CD69 up-regulation) and late (4-1BB expression) markers of T cell activation 3 and 15 hours after coculture initiation. For OT-I transgenic T cells, we find TCRβ down-regulation after 3 hours to be a high-fidelity readout of activation (Fig. 1B and fig. S2). This down-regulation reflects the internalization and degradation of the TCRαβ complex following strong affinity pMHC interaction, as previously described (fig. S2) (*28*). CD69 and 4-1BB expression, however, show slower kinetics; they not only require more time to be robustly expressed, but there is also evidence of TCR-independent CD69 expression possibly driven by cytokines and LPS in the culture, as has been previously documented (*29*–*32*). The distribution of Ca^2+^ concentration values, considering either all individual time points for all cells (left) or the average of each cell over the entire movie (right), shows a noticeable elevation in Ca^2+^ concentration only for antigen-specific T cells (Fig. 1C), while nonspecific T cells display baseline intracellular Ca^2+^ concentrations. Together, these results show that both the in vitro system and the analysis pipeline are appropriate.

Not all antigen-specific T cells up-regulate intracellular Ca^2+^ during the 2-hour imaging window. Because the development of an effective ML classifier, in principle, requires a high-quality training dataset, these non-activated antigen-specific T cells could potentially interfere with the performance of a predictive model. Therefore, we manually labeled the Ca^2+^ signals of each cell in the dataset as antigen-reactive or nonreactive based on visual inspection of the Indo-1 fluorescence ratio. Four independent evaluators blindly classified each cell based on its relative Ca^2+^ levels over time, and a majority vote determined the final assignment of reactivity status; 68.4% of all antigen-specific T cells and 4.11% of nonspecific T cells were labeled as antigen-reactive in the training datasets (Fig. 1D). This suggests that some Ca^2+^ fluctuation occurs in nonspecific T cells, although this would not ultimately result in productive activation (Fig. 1B). Using manual labeling as a method to classify T cell antigen specificity, we show a false discovery rate (*FDR*), i.e., the fraction of nonspecific T cells within all cells labeled as antigen-reactive, of 6.26% (Fig. 1E). Two unimodal distributions of average Ca^2+^ concentrations are observed on the basis of manual assignment of cell status as antigen-reactive or nonreactive. However, nonspecific cells manually labeled as antigen-reactive display an intermediate Ca^2+^ concentration distribution (Fig. 1F); while difficult for human evaluators to differentiate from antigen-specific T cells, it is possible that nonspecific cells with intracellular Ca^2+^ levels above baseline have Ca^2+^ fluctuation dynamics distinct from bona fide antigen-specific T cells. Appropriately trained ML algorithms should thus be able to distinguish these signaling events from TCR-dependent Ca^2+^ fluctuations. Last, we show a positive correlation between the activation efficiency of each independent culture well, determined by manual assignment of Ca^2+^ traces and molecular activation markers measured by flow cytometry, both at early (TCRβ: Pearson correlation coefficient, ρ = 0.976) and later (4-1BB: Pearson correlation coefficient, ρ = 0.649) time points (Fig. 1G), further validating the manual labeling process.

Increases in intracellular Ca^2+^ downstream of TCR engagement induce migration arrest (*13*, *33*–*36*). Given the importance of migration patterns in other approaches to identify T cell activation (*25*, *26*, *33*), we computed the average speed of all cells for each movie. We show that cells manually labeled as antigen-reactive are slower, on average, than those labeled as nonspecific (fig. S3A). In addition, at time points where Ca^2+^ concentration is low on antigen-reactive T cells (before activation), the average and instantaneous velocity is identical to or above that of nonspecific T cells (fig. S3, B and C).

### Deep learning approaches perform better than conventional methods for the classification of T cell activation based on Ca^2+^ fluctuations

We systematically tested a non-exhaustive list of ML models that have been extensively used for the classification of one-dimensional (1D) datasets. We divided all experiments into training and evaluation datasets, balancing the number antigen-specific and nonspecific T cells, as well as the number of cells manually labeled as antigen-reactive and nonreactive. Despite having confirmed that CTFR staining of either OT-I or P14 did not affect the critical parameters of this coculture setup (fig. S4), we also balanced the amount of movies with both CTFR staining conditions to prevent models from learning specific features of either condition (table S1). While the training datasets only contain cocultures of TCR-transgenic cells together with BMDCs presenting OVA, the test datasets consist of cocultures with either OVA or lymphocytic choriomeningitis virus gp33 (gp33-41) peptides to prevent overfitting and optimize the applicability of this model to a broader peptide repertoire. As expected, Ca^2+^ fluctuation of P14 T cells in gp33 cocultures is up-regulated as compared to their nonspecific OT-I TCR-transgenic counterparts (fig. S5). In addition, the manual labeling of the Ca^2+^ dynamics associated with gp33-stimulated T cell cocultures shows a similar *FDR* to the OVA cocultures (fig. S5).

To benchmark the algorithms and choose an optimal architecture, we computed the efficiency (fraction of cells correctly predicted as antigen-specific) and *FDR* (fraction of mispredicted cells) for each model. Assuming that a subset of antigen-specific T cells has not been activated, the model efficiency is calculated by comparing the prediction to the manual labels, rather than the genotype of the cell (OT-I or P14). This allows for evaluation of the efficiency of the ML model to predict antigen specificity independently of the efficiency of the in vitro model used to activate antigen-specific T cells. On the other hand, to generate a model that best predicts whether a T cell is antigen-specific, the *FDR* compares the prediction to the genotype (i.e., nonspecific T cells predicted as antigen-specific) for a measurement of accuracy. To compare models, the efficiency and accuracy metrics for both OVA and gp33 datasets are used to compute an ad hoc weighted performance metric, allowing a choice of the relative importance of accuracy over efficiency (see Materials and Methods); the model that maximizes this metric is chosen as optimal.

We first evaluated the use of a simple thresholding approach to classify each cell as either antigen-specific or nonspecific based on relative intracellular Ca^2+^ concentration. Setting a first threshold on intracellular Ca^2+^ levels separating time points with high ([Ca^2+^]^hi^) and low ([Ca^2+^]^lo^) average Ca^2+^ concentrations, we computed the time each cell spent in each Ca^2+^ state. A second threshold is then set; any cell spending more than this amount of time in the [Ca^2+^]^hi^ state was classified as antigen-specific (fig. S6A). For all possible pairs of thresholds, we computed the efficiency and accuracy of this method on the training dataset. The pair of thresholds that maximized the performance metric was then used to perform classification for the evaluation dataset (fig. S6B). This approach has high efficiency (95.2%) but relatively poor accuracy (*FDR* = 12.7%) for OVA cocultures (fig. S6C). Furthermore, this method is poorly applicable to cocultures where gp33 is used; despite good accuracy (*FDR* = 1.48%), a high percentage of antigen-specific cells were not predicted (70.4% efficiency), likely due to differences in average intracellular Ca^2+^ concentration between OVA- and gp33-specific TCR-transgenic T cells (fig. S6, C and D).

**Fig. 2.**
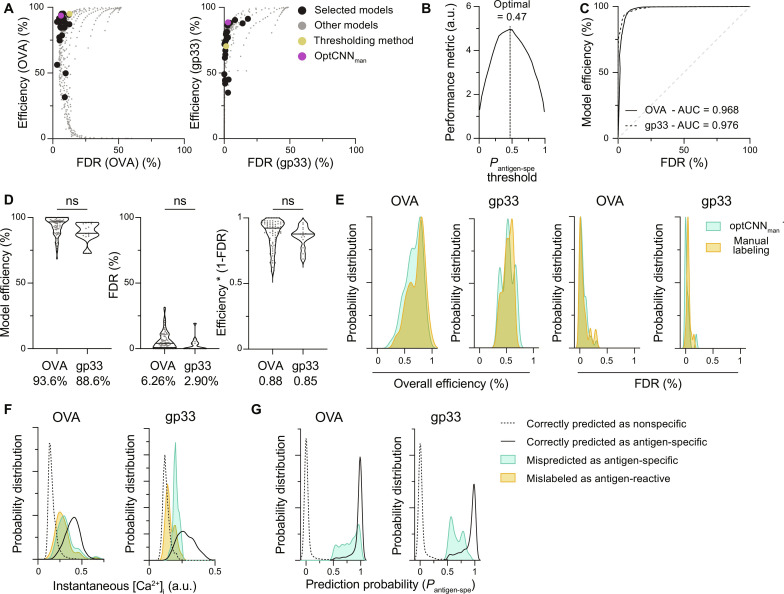
CNNs allow for efficient and accurate classification of T cell activation based on intracellular Ca^2+^ dynamics. (**A**) Model efficiency (frequency of cells manually labeled as antigen-reactive predicted as antigen-specific) and false discovery rate (*FDR*; frequency of nonreactive T cells predicted as antigen-specific) of all ML algorithms tested, for both OVA (left) and gp33 (right) time lapses. Performance of the selected models detailed in table S1 (black circles) is plotted along with the performance of the thresholding approach (fig. S6, B to D). Gray dots represent other models generated during the systematic evaluation of ML structures. The optimal optCNN_man_ model is shown in magenta. (**B**) Performance of optCNN_man_ (see Materials and Methods for the performance metrics) for varying thresholds (see fig. S6) on the probability of being antigen-specific (*P*_antigen-spe_). a.u., arbitrary units. (**C**) Receiver operating characteristic curve of optCNN_man_ for both OVA (full line) and gp33 (dotted line) time lapses. Area under the curve (AUC) represents the overall performance of optCNN_man_. (**D**) Detailed performance of optCNN_man_ using the prediction probability threshold of 0.47. Horizontal lines in the violin plot show the median and numbers below show the average of the distribution. Individual fields of view are represented in gray (*n* = 73 OVA and *n* = 15 gp33 fields of view over nine OVA and two gp33 independent experiments; Mann-Whitney *U* test). (**E**) Distribution of the overall efficiency (frequency of antigen-specific T cells predicted as antigen-specific) and *FDR* across the evaluation dataset for optCNN_man_ and the manual labeling process (*n* = 73 OVA and *n* = 15 gp33 fields of view over nine OVA and two gp33 independent experiments). (**F** and **G**) Distribution of intracellular Ca^2+^ concentration (F) and prediction probability *P*_antigen-spe_ (G) of the nonspecific cells mispredicted as antigen-specific (*n* = 174 OVA and *n* = 32 gp33 cells over nine OVA and two gp33 independent experiments). Not significant (ns), *P* > 0.05.

To find a more suitable approach for identifying antigen-specific cells based on intracellular Ca^2+^ levels, we tested a multitude of models for accuracy and efficiency, going from simpler to more complex architectures and assessing the need of preprocessing and data augmentation (Fig. 2A and table S2). Using the performance metric to choose an optimal model, we show that deep learning algorithms were generally superior to other ML approaches. In particular, CNN-based architectures performed much better than any other method with this dataset (fig. S6E), especially when the structure and training parameters are optimized (see Materials and Methods). The optimized CNN model using manually labeled cells as ground truth performs the best and is used for the rest of this study; it is referred to as optCNN_man_.

This systematic approach revealed several important insights. Normalization of calcium concentration across independent experimental days (see Materials and Methods) is a critical factor, improving the efficiency of prediction of gp33 time lapses by over 28% (table S2). Reevaluating the thresholding method with data normalization shows an improved performance to non-normalized data but still lags behind CNN (fig. S6F). Second, in this in vitro setup, as opposed to other similar studies, the addition of positional data (i.e., instantaneous cell speed) did not improve classification (table S2). Last, we observed that models trained with either the manual labels (T cells labeled as antigen-reactive versus labeled as nonreactive), the genotype (antigen-specific versus nonspecific T cells), or a combination of both (antigen-specific T cells labeled as antigen-reactive versus the rest) as ground truth, all perform relatively well (table S2). When the training parameters are optimized (table S3), all three models show a very similar performance (fig. S6F), and their predictions overlap for 94.5% of the cells in the evaluation dataset (12,421 of 13,145 cells) (fig. S6G). Hence, it appears, for this application, that this architecture is not very sensitive to contamination of the dataset by negative (non-activated OT-I) cells.

For each individual cell, optCNN_man_ provides a prediction probability; a threshold on this probability was used to determine the classification (Fig. 2B). The distribution of the probability of being antigen-specific (*P*_antigen-spe_) for all individual cells is bimodal, but classification of the rare cells that lie in between can markedly change the performance metrics. By varying the *P*_antigen-spe_ threshold, above which cells are predicted as antigen-specific, we show that the optimized architecture performs best when using the *P*_antigen-spe_ threshold of 0.47 (Fig. 2B).

The receiver operating characteristic curve shows the high sensitivity and specificity of the model with an area under the curve (AUC) of more than 0.95 (Fig. 2C). More specifically, optCNN_man_ has high efficiency for both OVA and gp33 cocultures (94.1 and 88.6%, respectively) and low error rates (6.26 and 2.90%, respectively) (Fig. 2D). To further facilitate comparisons of performance between datasets, we used the metric efficiency × (1 − *FDR*) for each individual field of view; this metric shows an overall performance that is nearly identical for both conditions (Fig. 2D). Furthermore, these predictions are similar to the predictions made by the human evaluators (Fig. 2E). The intracellular Ca^2+^ concentration of the nonspecific cells mispredicted as antigen-specific overlaps with those of nonspecific T cells manually labeled as antigen-reactive (Fig. 2F). Given the low prediction probability assigned to these cells (Fig. 2G), using a more restrictive threshold on *P*_antigen-spe_ would likely remove a large number of the false positive predictions, at the cost of reduced efficiency.

In an effort to validate the prediction algorithm at the single-cell level, i.e., by correlating the dynamics of Ca^2+^ fluctuation with indicators of T cell activation, we investigated motility changes in T cells predicted or not to be antigen-specific and as they relate to intracellular Ca^2+^ concentration. Using individual T cell trajectories from the analysis pipeline (Fig. 1A), we computed the instantaneous (between two frames) and the average (over the entire movie) velocity and compared cells predicted as antigen-specific to those predicted to be nonspecific. We show that, on average, antigen-specific T cells predicted as antigen-specific are slower than those predicted as nonspecific, as expected given their increased intracellular Ca^2+^ concentration (fig. S7, A and B) (*33*–*36*). At the single-cell level, we show that intracellular Ca^2+^ concentration and speed are inversely correlated, and a decrease in cell motility is detected as the cells presumably encounter cognate antigen (fig. S7C). We collated all 2443 cells predicted as antigen-specific by optCNNman and where we could identify the initial spike in intracellular Ca^2+^ concentration when this occurred during the imaging period. Comparing cell velocity with respect to this time point, we show that cell arrest is associated with an initial spike in intracellular Ca^2+^ concentration in cells predicted to be antigen-specific (fig. S7D).

### Biological validation of the Ca^2+^-based deep learning algorithm to predict antigen specificity

**Fig. 3.**
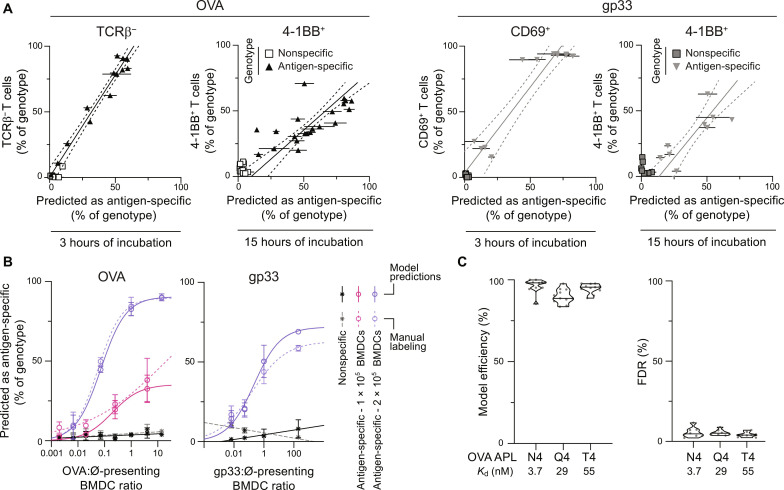
Biological validation of antigen specificity predictions based on intracellular Ca^2+^ dynamics. Critical parameters of the coculture (e.g., number of antigen-presenting BMDCs and total number of BMDCs) are modulated, and optCNN_man_ is used to predict the frequency of antigen-specific T cells. For each independent culture well, the prediction percentage is averaged over three fields of view; wells are then harvested and analyzed by flow cytometry for expression of selected activation markers. (**A**) Correlation between ML prediction and the frequency of cells down-regulating surface TCRβ expression or expressing CD69 or 4-1BB, as measured by flow cytometry, after 3 or 15 hours of incubation. Error bars indicate SEM. Linear regression of the antigen-specific conditions is shown (full line) with 95% confidence error (dotted lines) (TCRβ: *n* = 12 independent wells over three independent experiments, *R*^2^ = 0.959, ρ = 0.989; CD69: *n* = 12 independent wells over three independent experiments, *R*^2^ = 0.866, ρ = 0.930; 4-1BB and OVA peptide: *n* = 21 independent wells over four independent experiments, *R*^2^ = 0.493, ρ = 0.702; 4-1BB and gp33 peptide: *n* = 8 independent wells over four independent experiments, *R*^2^ = 0.746, ρ = 0.863). (**B**) Percentage of antigen-specific and nonspecific T cells predicted as antigen-specific as a function of the ratio of antigen-presenting to non-presenting BMDCs. Full lines and dotted lines represent a sigmoidal curve fitted to the data. The nonspecific group pools data from both 1 × 10^5^ and 2 × 10^5^ BMDC conditions. Error bars indicate SD. (**C**) Model efficiency (compared to manual labeling) and *FDR* of optCNNman when predicting T cell specificity to lower affinity antigens. Affinity (*K*_d_) of the altered peptide ligands (APLs) for the OT-I TCR is indicated (*37*) (*n* = 11 independent fields of view over three independent experiments).

We next sought to validate optCNN_man_ using an alternative approach. By altering the parameters of the BMDC:T cell coculture to modulate activation efficiency, we investigated how efficiently optCNN_man_ can predict activation in suboptimal conditions. To manipulate the antigen availability in each culture condition, we mixed antigen-presenting (OVA- or gp33-loaded) BMDCs and BMDCs presenting only endogenous peptides at different ratios. The lower absolute number of peptide-loaded APCs should lead to an increase in the time required for T cells to find cognate antigen, which translates, given the short imaging window, into a smaller fraction of activated cells. Using TCRβ down-regulation as well as CD69 and 4-1BB up-regulation after 3 and 15 hours of incubation as indicators of activation, we show a positive correlation between the cells predicted as antigen-specific by optCNN_man_ and the frequency of cells expressing these activation markers (Fig. 3A). Furthermore, we show, as anticipated, a dose-dependent relationship between the frequency of T cells predicted as antigen-specific and antigen availability (Fig. 3B). Similarly, decreasing the number of BMDCs in the coculture will reduce the probability of BMDC–T cell encounter and also leads to a reduction in the number of T cells predicted as antigen-specific (Fig. 3B).

Given that the training dataset was generated with a TCR-pMHC pair with high affinity (*K*_d_ = 3.7 ± 0.7 nM) (*37*), we sought to evaluate the effectiveness of optCNN_man_ for predicting the antigen specificity of T cells activated by lower affinity TCR-pMHC interactions. We stimulated naïve OT-I T cells with OVA (N4) altered peptide ligands (APLs; Q4 and T4) of decreasing affinity for the OT-I TCR (N4>Q4>T4) and keeping peptide concentration constant. We detected activation of OT-I T cells via flow cytometry, as indicated by CD69 and 4-1BB up-regulation after 3 and 15 hours, respectively (fig. S8A). As seen for stimulation of P14 T cells with gp33 (fig. S5A), lower avidity TCR engagement does not induce strong TCRβ down-regulation (fig. S8A). Despite subtle differences in the average intracellular calcium levels (fig. S8B), optCNN_man_ efficiently and accurately predicts antigen specificity over a wide range of physiologically relevant TCR-pMHC interactions (Fig. 3C).

**Fig. 4.**
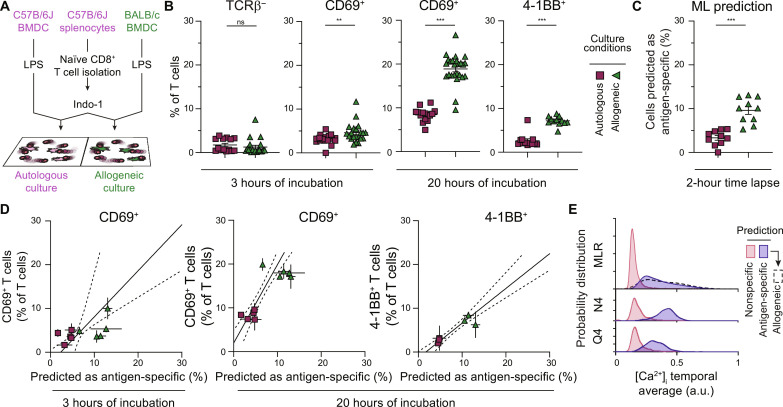
Deep learning models predict polyclonal T cell responses. (**A**) Schematic representation of the mixed lymphocyte reaction (MLR) setup. Purified C57B/6J naïve CD8^+^ T cells, stained with Indo-1, were overlaid on MHC-matched C57B/6J BMDCs (autologous condition) or MHC-mismatched BALB/c BMDCs (allogeneic condition). (**B**) Flow cytometry–based measurement of TCRβ, CD69, and 4-1BB expression 3 or 20 hours after coculture. Error bars show SD (*n* = 4 to 10 culture wells over three to five independent experiments; Mann-Whitney *U* test). (**C**) Frequency of cells predicted as antigen-specific (Mann-Whitney *U* test). (**D**) Correlation between ML prediction of antigen specificity and the frequency of cells expressing CD69 or 4-1BB (B), as measured by flow cytometry after 3 or 20 hours of incubation, respectively. Each point is the average of the prediction of three independent fields of view and the average of two to six independent culture wells for flow cytometry data. Error bars indicate SEM of prediction and flow cytometry data. Linear regression is shown (full line) with 95% confidence error (dotted lines) and its correlation coefficient (*R*^2^) and slope (three to five independent experiments). (**E**) Probability distribution of the average Ca^2+^ concentration over the entire time lapse for all the cells according to the prediction by optCNN_man_ and culture condition, comparing prediction for MLR cultures and monoclonal T cell culture with OVA N4 and OVA Q4 antigens (replotted from fig. S8) (MLR: *n* = 3219 nonspecific and *n* = 541 antigen-specific cells over five independent experiments; OVA: *n* = 7266 nonspecific and *n* = 3333 antigen-specific cells over three independent experiments; Q4: *n* = 1161 nonspecific and *n* = 972 antigen-specific cells over three independent experiments). a.u., arbitrary units. ns, *P* > 0.05; **P < 0.01; and ***P < 0.005.

Because it is not possible to know a priori the antigen specificity of individual naïve polyclonal CD8^+^ T cells, the validation of the model on polyclonal responses to antigenic peptides is challenging without extensive experimental confirmation. Thus, we used a mixed lymphocyte reaction (MLR) that typically leads to a larger fraction of T cells being activated in a polyclonal fashion as compared to antigen-specific T cells. We cocultured C57BL/6J CD8^+^ T cells with MHC-matched C57BL/6J (autologous) or MHC-mismatched BALB/c (allogeneic) BMDCs (Fig. 4A). Using molecular markers of activation measured by flow cytometry after 3 and 20 hours, we show that allogeneic culture conditions lead to a higher fraction of T cells expressing activation markers than in autologous conditions. Although apparent as early as 3 hours, monitoring of activation by flow cytometry is more efficient after 20 hours of coculture (Fig. 4B and fig. S9). Cell surface TCRβ down-regulation is not an obvious marker of T cell activation in the MLR setting (Fig. 4B). Using optCNN_man_ to predict T cell activation based on Ca^2+^ fluctuations in this polyclonal system, we also show that T cells cultured in allogeneic culture conditions have a higher frequency of cells predicted as antigen-specific than when T cells are cultured with autologous BMDCs (Fig. 4C). In addition, there is a strong correlation between the ML predictions and the flow cytometry markers of activation, particularly after 20 hours of culture, confirming the accuracy of prediction (Fig. 4D). The distribution of intracellular Ca^2+^ concentrations in polyclonal T cells predicted as antigen-specific in the MLR is much wider than that of the monoclonal T cell populations used earlier (Fig. 4E), with an average calcium concentration closer to that of Q4-stimulated OT-I; this may be due to the wider range of affinities in the polyclonal T cell repertoire and differences in alloreactive TCR-pMHC binding biomechanics (*38*).

Together, these data show the applicability of optCNN_man_, trained on monoclonal T cells responding to a single high-affinity peptide, for the prediction of responses to additional peptides as well as polyclonal T cell responses. Thus, simple models of T cell activation can be used to train ML architectures to recognize general features of Ca^2+^ fluctuation, which are common to T cell responses across a wider range of TCR-pMHC affinities.

## DISCUSSION

The rapid identification of antigen-specific T cells from naïve polyclonal T cells presents a unique challenge due to the lack of reliable early markers of TCR-specific T cell activation before proliferation. Here, we demonstrate the feasibility of using the time-dependent fluctuations of intracellular Ca^2+^ concentration in individual T cells, a TCR-proximal readout, as a means to identify their antigen specificity. While increases in intracellular Ca^2+^ may not be strictly TCR-specific, we propose that ML algorithms, trained on T cells of known specificity and activated in an antigen-specific manner, can learn the features of Ca^2+^ fluctuation associated with TCR-pMHC engagement. We show that, once trained on monoclonal T cell responses, this model can be applied to predict activation of T cells over a relatively broad range of TCR-pMHC affinities and polyclonal T cells. We believe that this approach could be used to predict the antigen specificity of polyclonal T cells activated with specific peptides or peptide pools. The performance of deep learning models, once trained, is independent of the frequency of the event being predicted, i.e., antigen specificity predictions for T cells at a 1:1 antigen-specific:nonspecific ratio (as shown here) will be as efficient as at the much lower ratio of antigen-specific T cells in the polyclonal T cell population.

As compared to other methods of antigen-specific T cell identification, we suggest that the monitoring of intracellular Ca^2+^ signaling is a much faster and simpler approach to the identification of antigen-specific T cells. Although they do not require T cell stimulation, thereby bypassing the delay in the modulation of activation marker expression, multimer pMHC-based enrichment requires the engineering of a new reagent for each peptide and every individual peptide/MHC combination, some of which may be problematic to manufacture (*39*). Early T cell activation is also challenging to measure by flow cytometry due to the absence of appropriate markers. We show that, while early (3 hours) surface TCRβ down-regulation and CD69 expression are useful for select TCRs in the in vitro model used here, they were not useful in the more physiologically relevant polyclonal MLR cultures and for lower avidity TCR-pMHC interactions. 4-1BB, although more specific, appears to be up-regulated much later and only maximally after some proliferation has occurred, limiting its usefulness for early isolation of antigen-specific T cells and biased by preferential expansion of high-affinity T cell clones. In comparison, increases in intracellular Ca^2+^, a very early indicator of the TCR signaling pathway, allows for rapid, within a 2-hour time frame, and accurate identification of antigen-specific T cells. The simple nature of the coculture setup, commonly used for antigen-specific T cell activation, allows for flexibility in the target antigen (e.g., cancer antigens) loaded and MHC/HLA-restriction, by varying the source of APCs. It is important to point out that the dynamics of Ca^2+^ fluctuations generated observed with this coculture system are not necessarily representative of those observed with physiological models (e.g., lower peptide concentration or in vivo activation). The decision to use high concentrations of peptides was based on the goal of optimizing antigen-specific T cell discovery rather than mimicking physiological Ca^2+^ dynamics; high concentrations of peptide, particularly in the case of low affinity interactions, allow for broader activation of T cells.

Recent studies have also investigated the use of imaging-based technologies and ML to predict antigen specificity, analyzing either the dynamics of interaction between antigen-specific T cells and APCs or the changes in metabolic state associated with pan–T cell activation (*25*–*27*). With an AUC of more than 0.95, the performance of the approach presented here is similar to, if not better than, these previously published studies. In addition, the stimulation of naïve CD8^+^ T cells with peptide-loaded BMDCs, rather than pan T cell stimulation, ensures that the ML models learn features of Ca^2+^ fluctuation, which are generated during TCR engagement. This study also demonstrates the ability to predict T cell reactivity across a range of TCR-pMHC affinities.

In terms of experimental complexity, the use of simple, inexpensive, and widely accessible fluorescent dyes and labware for conventional fluorescence microscopes are the only requirements and represent a low cost of entry for using this technology for downstream applications. The use of the ratiometric Indo-1 dye rather than genetically encoded reporters facilitates the application of these technique to a much wider variety of monoclonal T cell models and translation to the human system. Extraction of Ca^2+^ fluctuations from these movies and the training of the 1D ML network also has the major advantage of requiring very little computing power and can be replicated with any desktop computer. Here, we made use of naïve T cells for the evaluation of Ca^2+^ fluctuations. Naïve T cells, as opposed to antigen-experienced T cells, are not restricted in their TCR repertoire as may be the case after clonal expansion and may allow for the identification of TCRs with a wider range of affinities for a peptide of interest. Furthermore, naïve and antigen-experienced T cells and even different antigen-experienced T cell subsets display distinct Ca^2+^ fluctuation patterns in response to TCR stimulation (*40*, *41*). The use of purified naïve T cells, although themselves heterogeneous in nature (*42*), should allow for a more homogenous and reproducible Ca^2+^ response to TCR stimulation, compared to antigen-experienced T cells.

In this proof-of-concept study, we demonstrated that performant ML algorithms can be trained on Ca^2+^ fluctuations in activated monoclonal T cells to predict polyclonal T cell responses, using a limited amount of data (~10,000 cells). Substantially increasing the size of the dataset with additional time lapses or through AI-assisted methods may further improve model performance, especially when it comes to differentiating the distinct pattern of Ca^2+^ fluctuation associated with nonspecific T cells mispredicted as antigen-specific from bona fide antigen-specific T cells (*43*–*45*). Furthermore, it has been previously demonstrated that Ca^2+^ oscillations contain information about the affinity of TCR-pMHC interactions; lower affinity TCR engagement will typically lead to more transient Ca^2+^ fluctuations and distinct early activation dynamics (*14*–*16*). Using a similar approach, we postulate that generating Ca^2+^ fluctuations from monoclonal T cells following TCR stimulation with pMHC of varying affinity at lower peptide concentrations should enable training of ML models to recognize specific features of Ca^2+^ fluctuation associated with low versus high affinity TCR binding to antigen. It has already been shown that an ML model, trained on the dynamics of cytokine release by T cells following stimulation over several weeks, can predict the antigen affinity of individual T cells (*46*). The use of intracellular Ca^2+^ dynamics would fast-track and simplify this approach. Notably, in the experiments presented here, we observed only modest differences in Ca^2+^ fluctuations when OT-I T cells were activated with APLs. However, it is important to note that the high peptide concentration used in this study, while optimizing the identification of low affinity antigen-specific cells, may mask differences in Ca^2+^ fluctuations between different stimulation conditions.

Combining ML approaches for the identification of antigen-specific T cells with technologies for their isolation will allow for the isolation of T cells of interest for downstream single-cell TCR-sequencing, identifying clinically relevant TCR sequences for use in adoptive therapy. Few methods allow for the isolation of single cells of interest following microscopy-based observations in a high-throughput and automated fashion. A few studies have demonstrated the use of microfluidics, micropipettes, and/or a microraft apparatus for the isolation of antigen-specific T cells that may also be challenging to manufacture and are relatively low-throughput technologies (*19*, *47*–*50*). However, we and others have recently described methods to tag and/or isolate cells with high specificity under the microscope using the targeted illumination of cells of interest (*51*–*55*). Particular attention will need to be paid to the compounding of errors at the various steps of the pipeline (tracking, ML prediction, and barcoding) to avoid contamination by nonspecific TCR sequences, which would increase the time required for downstream biological validation of identified TCR sequence. The weights *w*_*eff*_ and *w*_*FDR*_ used in the performance metric, used to select the optimal model for antigen-specific T cell prediction, can be adjusted to choose optimal models to reduce *FDR* or increase the efficiency of identification, depending on the downstream applications. However, the advent of fast and reliable in vitro and in silico pipelines for TCR-pMHC screening mitigates this risk (*56*, *57*). Furthermore, the possibility to identify TCR sequences with a specific affinity, fine-tuned either for acute antitumor activity (high affinity) or for longer-lasting, broader immune-surveillance, with reduced side effects (lower affinity) could facilitate improvement in the quality of care to patients requiring adoptive T cell therapy (*7*, *9*).

## MATERIALS AND METHODS

### Mice

C57BL/6J (RRID:IMSR_JAX:000664) and BALB/c (RRID:IMSR_JAX:000651) mice were purchased from the Jackson Laboratory (Bar Harbor, ME, USA). C57BL/6J-Tg(OT-I)-Rag1<tm1Mom> (OT-I, RRID:IMSR_JAX:003831) mice were obtained through the National Institute of Allergy and Infectious Diseases Exchange Program, National Institutes of Health (Bethesda, MD, USA) (*58*, *59*). P14 TCR Tg mice were provided by M. Richer (McGill University, Montreal, Canada) and crossed onto a TCRα knockout (KO) (the Jackson Laboratory, stock no. 002116) background (RRID:MMRRC_037394-JAX) (*60*, *61*). All mice were bred and maintained in specific pathogen–free animal facilities at the Maisonneuve-Rosemont Hospital Research Centre and the Comparative Medicine and Animal Resource Center at McGill University. Both male and female mice 6 to 12 weeks of age were used. All animal protocols have been approved by the Animal Care Committee at the Maisonneuve-Rosemont Hospital Research Centre and McGill University. Experiments were performed in accordance with the Canadian Council on Animal Care guidelines.

### In vitro T cell coculture assay

Bone marrow cells (1 × 10^6^), harvested from the indicated mice (male and female, 6 to 12 weeks old), are plated in six-well adherent plates for 7 days in 4 ml of 10% fetal bovine serum (HyClone, catalog no. SH30396.03), 100 mM Hepes (Multicell, catalog no. 330-050-EL), 100 IU of penicillin/streptomycin (Multicell, catalog no. 450-201-EL), 1 mM sodium pyruvate (Multicell, catalog no. 600-110-EL), 0.1 mM MEM nonessential amino acids (Gibco, catalog no. 11140-050), and 2 mM l-glutamine RPMI 1640 (Multicell, catalog no. 350-000-CL). Culture medium is supplemented with 1000 U of murine granulocyte-macrophage colony-stimulating factor per well (BioLegend, catalog no. 576302) and a predetermined dose of P815-IL4 supernatant. Supplemented medium (2 ml) is replaced after 2 and 3 days of culture. At day 6, 4 μM OVA N4 (OVA 257-264; AnaSpec Inc., catalog no. AS-60193-5), gp33 (gp33-41; AnaSpec Inc., catalog no. AS-61669), OVA Q4 (OVA 257-264 Q4 variant; AnaSpec Inc., catalog no. AS-64402), or OVA T4 (OVA 257-264 T4 variant; AnaSpec Inc., catalog no. AS-64403) peptide and LPS (1 μg/ml) are added to the culture. At day 7, 8, or 9, BMDCs are harvested from culture and enriched using a 14.7% Histodenz (Sigma-Aldrich, catalog no. D2158) gradient.

For T cell isolation, cellular suspension is harvested via physical dissociation from OT-I and P14 spleen and lymph nodes (male and female, 6 to 12 weeks old). Naïve CD8^+^ T cells are further isolated using a magnetic enrichment kit according to the manufacturer’s specifications (STEMCELL Technologies, catalog no. 19858). OT-I or P14 cells are stained with 2 μM CTFR (Invitrogen, catalog no. C34572) at 10^6^ cells/ml for 15 min at 37°C and rested 15 min at 37°C, 5% CO_2_ before being pooled. Unless otherwise stated, OT-I and P14 are mixed at a 1:1 ratio. The cell suspension is then stained with 10 μM Indo-1 for 30 min at 37°C and rested 30 min at 37°C, 5% CO_2_. The isolation and staining procedure are identical for C57B/6J and BALB/c naïve CD8^+^ T cells, but the T cells are kept separate at all times and are not stained with CTFR. For MLR experiments, sex-matched T cells and BMDCs are cocultured to avoid anti-Sex determining Region Y (SRY) immune responses.

Right before imaging, unless otherwise specified, 2 × 10^5^ BMDCs and 2 × 10^5^ T cells are pooled in phenol red–free imaging medium [10% fetal bovine product (HyClone, catalog no. SH30109.03) and 100 IU of penicillin/streptomycin)] and plated onto fibronectin-coated (2 μg/cm^2^; Sigma-Aldrich, catalog no. F2006) 18-well slides (ibidi, catalog no. 81816) for imaging. Wide-field epifluorescence images of Indo-1 (405 and 447 nm) and CTFR (698 nM) are captured every 30 s (Indo-1) or 10 min (CTFR) for 2 hours on a Nikon Eclipse Ti2, under a stage top incubator, with mercury lamp illumination. After imaging, slides are kept in an incubator before harvesting for flow cytometric analysis.

### Flow cytometric analysis

Flow cytometry analysis of mouse surface antigens was performed with the following antibodies: anti-CD3ε (145-2C11, catalog no. 100328, RRID:AB_893318, 1:100), anti-CD8α (53-6.7, catalog no. 100714, RRID:AB_312753, 1:400), anti-CD11c (N418, catalog no. 117308, RRID: AB_313776, 1:1600), anti-CD45.2 (104, catalog no. 109822, RRID:AB_493731, 1:100), anti-CD80 (16-10A1, catalog no. 104722, RRID:AB_2291392, 1:400), anti–MHC class II (I-A/I-E) (M5/114.15.2, catalog no. 107630, RRID:AB_2069376, 1:1600), anti-TCRβ (H57-597, catalog no. 109224, RRID:AB_1027648, 1:100), anti-NK1.1 (PK136, catalog no. 108706, RRID:AB_313393, 1:200), anti-CD19 (6D5, catalog no. 115505, RRID:AB_313641, 1:200), anti-CD11b (M1/70, catalog no. 101206, RRID:AB_312788, 1:200), anti-TCRγδ (N418, catalog no. 117306, RRID:AB_313775, 1:200), anti-CD44 (IM7, catalog no. 103012, RRID:AB_312962, 1:200), anti-CD69 (H1.2F3, catalog no. 104522, RRID:AB_2260065, 1:100), anti-CD137 (17B5, catalog no. 106105, RRID:AB_2287565, 1:200), anti-TCR Vα2 (B20.1, catalog no. 127807 RRID:AB_1134184, 1:100), anti-TCR Vβ5 (MR9-4, catalog no. 139507, RRID:AB_2566021, 1:100), and Zombie Aqua (catalog no. 423101) or Green fixable viability dye (catalog no. 423111) (BioLegend). Staining was performed for 20 min at 4°C. Flow cytometry analyses were performed on a LSR Fortessa X20, and data were analyzed using FlowJo software (BD Biosciences).

### Prediction of T cell antigen specificity

All the code used for the prediction of T cell specificity was coded using MATLAB (MathWorks).

#### In silico analysis pipeline

From raw images, segmentation and tracking of T cells are made by adapting previously published methods (*62*–*64*). Briefly, in each frame, the centroid of each T cell is localized from the sum of the Indo-1 images using a combination of edge detection and watershed segmentation methods. On the basis of the position of all cells at each time point, we calculate individual T cell trajectories using particle tracking–based methods, adapted for our particular application. Filtering of tracks based on length (at least half of the duration of the movie) ensures that all cells in a time lapse are independent from each other. The distance traveled by a cell between two frames is reported as instantaneous speed. Manual quality control on the tracking is made to remove mistracked cells.

For each cell, at each time point, the fluorescence intensity of both Indo-1 emission wavelengths is calculated by integrating pixel intensity values in a disk (60% of the cell’s diameter) around the centroid. The intensity background (calculated locally for each cell) is subtracted to the fluorescence intensity before calculating the ratio of both wavelengths (405/447 nm). Assembling the ratio for each cell across all time points allows us to generate the intracellular Ca^2+^ dynamics. For genotype assignment, the fluorescence of CTFR is obtained 12 times throughout the imaging period for each cell; cell type is assigned if, at least eight time points, the cell appears positive or negative for CTFR.

For manual labeling, four independent evaluators were shown the intracellular Ca^2+^ dynamics of all cells and were asked to classify them as antigen-reactive or nonreactive. A majority vote between evaluators was used to determine the final label of each cell.

#### Training and evaluation of the ML algorithms

For all individual models, training was made on the training dataset using the 1D intracellular Ca^2+^ signal as input and the genotype of each cell, their manual label, or a combination of both (antigen-specific T cells manually labeled as antigen-reactive) as ground truth. When indicated, Ca^2+^ dynamics were complemented either with the derivative of the Ca^2+^ dynamics, approximated by the absolute value of the difference in Ca^2+^ levels between two consecutive time points, or with the instantaneous cell speed, approximated by the euclidean distance between the same cell at two consecutive time points. Evaluation of the model was made on the evaluation dataset by predicting antigen specificity for each individual time lapse (field of view).

#### Performance metric

For each time lapse, the model efficiency, overall efficiency, and *FDR* were measured as follows

$$\text{Model efficiency}=\frac{\#\;\text{of antigen-specific T cells predicted as antigen-specific}}{\#\;\text{of antigen-specific T cells manually labeled antigen-specific}}$$ (1)

$$\text{Overall efficiency}=\frac{\#\;\text{of antigen-specific T cells predicted as antigen-specific}}{\#\;\text{of antigen-specific T cells}}$$ (2)

$$\text{False discovery rate}=\frac{\#\;\text{of nonspecific T cells predicted as antigen-specific}}{\#\;\text{of T cells manually labeled as antigen-specific}}$$ (3)

All throughout, the performance metric (*pM*) uses the average model efficiency (*eff*) and the average *FDR* across all time lapses in the evaluation dataset. The optimal model is the one that maximized the formula

$$\text{pM}=\frac{1}{\sqrt{W_{\text{eff}}\;\times \;{\left(1-{\text{eff}}_{\text{OVA}}\right)}^{2}+W_{\text{eff}}\;\times \;{\left(1-{\text{eff}}_{\text{gp}33}\right)}^{2}+W_{\text{FDR}}{\;\times \;(0-{\text{FDR}}_{\text{OVA}})}^{2}+W_{\text{FDR}}\;\times \;{\left(0-{\text{FDR}}_{\text{OVA}}\right)}^{2}}}$$ (4)

*w*_*eff*_ and *w*_*FDR*_ are variable parameters that modulate the importance attributed to the error rate or the efficiency for each particular application. For this study, we use *w*_*eff*_ = 1 and *w*_*FDR*_ = 5.

#### Data normalization

When indicated, Ca^2+^ concentration of each cell was normalized to the average value of “resting” intracellular Ca^2+^ concentration. Briefly, for each time lapse, two Gaussian distributions are fitted to the probability distribution function of intracellular Ca^2+^ calcium concentration. The mean of each Gaussian distribution is used as the average value Ca^2+^ concentration in the low ([Ca^2+^]^lo^) and high ([Ca^2+^]^hi^) state for this movie. For each cell at each time point, we divide its Ca^2+^ concentration by the average [Ca^2+^]^lo^ value to compute the “fold change” of Ca^2+^ concentration over the resting state, as a means to reduce inter-experiment variability.

#### Data augmentation procedure

When indicated, data augmentation was performed by artificially generating in silico Ca^2+^ fluctuations from real in vitro fluctuations. This is achieved by a combination of repeating existing data, adding noise to the existing data, shifting the start of Ca^2+^ fluctuation forward or backward (in time) and increasing or decreasing the levels of the Ca^2+^ in the existing data.

#### Hyperoptimization

For each parameter to be optimized, i.e., number of neurons, kernel size, optimizer, and mini batch size, a range of possibilities was determined according to commonly used values for that parameter in the literature. A model was trained and evaluated as previously described for each combination of these four parameters, across all the ranges. All the models were then evaluated using the weighted performance metric; the hyperoptimized model is the one maximizing the performance metric.

### Statistical analysis

Unless otherwise stated, a two-sample nonparametric Mann-Whitney *U* test was performed using Prism (GraphPad).

## References

[1] P. Shafer, L. M. Kelly, V. Hoyos, Cancer therapy with TCR-engineered T cells: Current strategies, challenges, and prospects. Front. Immunol. 13, 835762 (2022). 35309357 10.3389/fimmu.2022.835762 PMC8928448

[2] E. Baulu, C. Gardet, N. Chuvin, S. Depil, TCR-engineered T cell therapy in solid tumors: State of the art and perspectives. Sci. Adv. 9, eadf3700 (2023). 36791198 10.1126/sciadv.adf3700 PMC9931212

[3] D. Hudson, R. A. Fernandes, M. Basham, G. Ogg, H. Koohy, Can we predict T cell specificity with digital biology and machine learning? Nat. Rev. Immunol. 23, 511–521 (2023). 36755161 10.1038/s41577-023-00835-3 PMC9908307

[4] Q. Li, Z.-Y. Ding, The ways of isolating neoantigen-specific T cells. Front. Oncol. 10, 1347 (2020). 32850430 10.3389/fonc.2020.01347 PMC7431921

[5] S. Zhong, K. Malecek, L. A. Johnson, Z. Yu, E. Vega-Saenz de Miera, F. Darvishian, K. McGary, K. Huang, J. Boyer, E. Corse, Y. Shao, S. A. Rosenberg, N. P. Restifo, I. Osman, M. Krogsgaard, T-cell receptor affinity and avidity defines antitumor response and autoimmunity in T-cell immunotherapy. Proc. Natl. Acad. Sci. U.S.A. 110, 6973–6978 (2013). 23576742 10.1073/pnas.1221609110 PMC3637771

[6] M. Hebeisen, M. Allard, P. O. Gannon, J. Schmidt, D. E. Speiser, N. Rufer, Identifying individual T cell receptors of optimal avidity for tumor antigens. Front. Immunol. 6, 582 (2015). 26635796 10.3389/fimmu.2015.00582 PMC4649060

[7] E. D’Ippolito, K. Schober, M. Nauerth, D. H. Busch, T cell engineering for adoptive T cell therapy: Safety and receptor avidity. Cancer Immunol. Immunother. 68, 1701–1712 (2019). 31542797 10.1007/s00262-019-02395-9 PMC11028346

[8] M. M. Hoffmann, J. E. Slansky, T-cell receptor affinity in the age of cancer immunotherapy. Mol. Carcinog. 59, 862–870 (2020). 32386086 10.1002/mc.23212 PMC7340130

[9] D. Campillo-Davo, D. Flumens, E. Lion, The quest for the best: How TCR affinity, avidity, and functional avidity affect TCR-engineered T-cell antitumor responses. Cell 9, 1720 (2020). 10.3390/cells9071720 PMC7408146 32708366

[10] J. Schmidt, J. Chiffelle, M. A. S. Perez, M. Magnin, S. Bobisse, M. Arnaud, R. Genolet, J. Cesbron, D. Barras, B. Navarro Rodrigo, F. Benedetti, A. Michel, L. Queiroz, P. Baumgaertner, P. Guillaume, M. Hebeisen, O. Michielin, T. Nguyen-Ngoc, F. Huber, M. Irving, S. Tissot-Renaud, B. J. Stevenson, S. Rusakiewicz, D. Dangaj Laniti, M. Bassani-Sternberg, N. Rufer, D. Gfeller, L. E. Kandalaft, D. E. Speiser, V. Zoete, G. Coukos, A. Harari, Neoantigen-specific CD8 T cells with high structural avidity preferentially reside in and eliminate tumors. Nat. Commun. 14, 3188 (2023). 37280206 10.1038/s41467-023-38946-z PMC10244384

[11] M. Shakiba, P. Zumbo, G. Espinosa-Carrasco, L. Menocal, F. Dündar, S. E. Carson, E. M. Bruno, F. J. Sanchez-Rivera, S. W. Lowe, S. Camara, R. P. Koche, V. P. Reuter, N. D. Socci, B. Whitlock, F. Tamzalit, M. Huse, M. D. Hellmann, D. K. Wells, N. A. Defranoux, D. Betel, M. Philip, A. Schietinger, TCR signal strength defines distinct mechanisms of T cell dysfunction and cancer evasion. J. Exp. Med. 219, (2022). 10.1084/jem.20201966 PMC8704919 34935874

[12] M. N. Duong, E. Erdes, M. Hebeisen, N. Rufer, Chronic TCR-MHC (self)-interactions limit the functional potential of TCR affinity-increased CD8 T lymphocytes. J. Immunother. Cancer 7, 284 (2019). 31690351 10.1186/s40425-019-0773-z PMC6833194

[13] K. S. Friedmann, M. Bozem, M. Hoth, Calcium signal dynamics in T lymphocytes: Comparing in vivo and in vitro measurements. Semin. Cell Dev. Biol. 94, 84–93 (2019). 30630031 10.1016/j.semcdb.2019.01.004

[14] B. Dura, S. K. Dougan, M. Barisa, M. M. Hoehl, C. T. Lo, H. L. Ploegh, J. Voldman, Profiling lymphocyte interactions at the single-cell level by microfluidic cell pairing. Nat. Commun. 6, 5940 (2015). 25585172 10.1038/ncomms6940

[15] A. Salles, C. Billaudeau, A. Sergé, A.-M. Bernard, M.-C. Phélipot, N. Bertaux, M. Fallet, P. Grenot, D. Marguet, H.-T. He, Y. Hamon, Barcoding T cell calcium response diversity with methods for automated and accurate analysis of cell signals (MAAACS). PLOS Comput. Biol. 9, e1003245 (2013). 24086124 10.1371/journal.pcbi.1003245 PMC3784497

[16] M. Le Borgne, S. Raju, B. H. Zinselmeyer, V. T. Le, J. Li, Y. Wang, M. J. Miller, A. S. Shaw, Real-time analysis of calcium signals during the early phase of T Cell activation using a genetically encoded calcium biosensor. J. Immunol. 196, 1471–1479 (2016). 26746192 10.4049/jimmunol.1502414 PMC4744592

[17] S. N. Christo, K. R. Diener, R. E. Nordon, M. P. Brown, H. J. Griesser, K. Vasilev, F. C. Christo, J. D. Hayball, Scrutinizing calcium flux oscillations in T lymphocytes to deduce the strength of stimulus. Sci. Rep. 5, 7760 (2015). 25585590 10.1038/srep07760 PMC4293621

[18] N. Anikeeva, D. Grosso, N. Flomenberg, Y. Sykulev, Evaluating frequency and quality of pathogen-specific T cells. Nat. Commun. 7, 13264 (2016). 27786275 10.1038/ncomms13264 PMC5095286

[19] A. I. Segaliny, G. Li, L. Kong, C. Ren, X. Chen, J. K. Wang, D. Baltimore, G. Wu, W. Zhao, Functional TCR T cell screening using single-cell droplet microfluidics. Lab Chip 18, 3733–3749 (2018). 30397689 10.1039/c8lc00818c PMC6279597

[20] S. Wang, Y. Liu, Y. Li, M. Lv, K. Gao, Y. He, W. Wei, Y. Zhu, X. Dong, X. Xu, Z. Li, L. Liu, Y. Liu, High-throughput functional screening of antigen-specific T cells based on droplet microfluidics at a single-cell level. Anal. Chem. 94, 918–926 (2022). 34852202 10.1021/acs.analchem.1c03678

[21] N. Sapoval, A. Aghazadeh, M. G. Nute, D. A. Antunes, A. Balaji, R. Baraniuk, C. J. Barberan, R. Dannenfelser, C. Dun, M. Edrisi, R. A. L. Elworth, B. Kille, A. Kyrillidis, L. Nakhleh, C. R. Wolfe, Z. Yan, V. Yao, T. J. Treangen, Current progress and open challenges for applying deep learning across the biosciences. Nat. Commun. 13, 1728 (2022). 35365602 10.1038/s41467-022-29268-7 PMC8976012

[22] M. Pertseva, B. Gao, D. Neumeier, A. Yermanos, S. T. Reddy, Applications of machine and deep learning in adaptive immunity. Annu. Rev. Chem. Biomol. Eng. 12, 39–62 (2021). 33852352 10.1146/annurev-chembioeng-101420-125021

[23] P. Rajpurkar, E. Chen, O. Banerjee, E. J. Topol, AI in health and medicine. Nat. Med. 28, 31–38 (2022). 35058619 10.1038/s41591-021-01614-0

[24] G. Rong, A. Mendez, E. B. Assi, B. Zhao, M. Sawan, Artificial intelligence in healthcare: Review and prediction case studies. Proc. Est. Acad. Sci. Eng. 6, 291–301 (2020).

[25] P. N. Anandakumaran, A. G. Ayers, P. Muranski, R. J. Creusot, S. K. Sia, Rapid video-based deep learning of cognate versus non-cognate T cell-dendritic cell interactions. Sci. Rep. 12, 559 (2022). 35017558 10.1038/s41598-021-04286-5 PMC8752671

[26] V. M. Liarski, A. Sibley, N. van Panhuys, J. Ai, A. Chang, D. Kennedy, M. Merolle, R. N. Germain, M. L. Giger, M. R. Clark, Quantifying in situ adaptive immune cell cognate interactions in humans. Nat. Immunol. 20, 503–513 (2019). 30778242 10.1038/s41590-019-0315-3 PMC6474677

[27] A. J. Walsh, K. P. Mueller, K. Tweed, I. Jones, C. M. Walsh, N. J. Piscopo, N. M. Niemi, D. J. Pagliarini, K. Saha, M. C. Skala, Classification of T-cell activation via autofluorescence lifetime imaging. Nat Biomed Eng. 5, 77–88 (2021). 32719514 10.1038/s41551-020-0592-z PMC7854821

[28] M. H. Lee, D. Min, C. H. Sonn, K. N. Lee, K. E. Kim, S. G. Paik, Y. S. Kim, TCR internalization induced by peptide/MHC ligands requires the transmembrane domains of alphabeta chains of TCR, but not the expression of CD8 and Thy-1 molecules. Mol. Cells 9, 617–624 (1999). 10672928

[29] K.-M. Kim, H. W. Kim, J.-O. Kim, K.-M. Baek, J. G. Kim, C.-Y. Kang, Induction of 4-1BB (CD137) expression by DNA damaging agents in human T lymphocytes. Immunology 107, 472–479 (2002). 12460192 10.1046/j.1365-2567.2002.01538.x PMC1782822

[30] B. E. Freeman, E. Hammarlund, H.-P. Raué, M. K. Slifka, Regulation of innate CD8+ T-cell activation mediated by cytokines. Proc. Natl. Acad. Sci. U.S.A. 109, 9971–9976 (2012). 22665806 10.1073/pnas.1203543109 PMC3382521

[31] L. R. Shiow, D. B. Rosen, N. Brdicková, Y. Xu, J. An, L. L. Lanier, J. G. Cyster, M. Matloubian, CD69 acts downstream of interferon-alpha/beta to inhibit S1P1 and lymphocyte egress from lymphoid organs. Nature 440, 540–544 (2006). 16525420 10.1038/nature04606

[32] D. F. Tough, S. Sun, J. Sprent, T cell stimulation in vivo by lipopolysaccharide (LPS). J. Exp. Med. 185, 2089–2094 (1997). 9182680 10.1084/jem.185.12.2089 PMC2196347

[33] T. X. Dong, S. Othy, M. L. Greenberg, A. Jairaman, C. Akunwafo, S. Leverrier, Y. Yu, I. Parker, J. L. Dynes, M. D. Cahalan, Intermittent Ca2+ signals mediated by Orai1 regulate basal T cell motility. eLife 6, e27827 (2017). 29239723 10.7554/eLife.27827 PMC5747518

[34] E. Donnadieu, G. Bismuth, A. Trautmann, Antigen recognition by helper T cells elicits a sequence of distinct changes of their shape and intracellular calcium. Curr. Biol. 4, 584–595 (1994). 7953532 10.1016/s0960-9822(00)00130-5

[35] P. A. Negulescu, T. B. Krasieva, A. Khan, H. H. Kerschbaum, M. D. Cahalan, Polarity of T cell shape, motility, and sensitivity to antigen. Immunity 4, 421–430 (1996). 8630728 10.1016/s1074-7613(00)80409-4

[36] N. R. Bhakta, D. Y. Oh, R. S. Lewis, Calcium oscillations regulate thymocyte motility during positive selection in the three-dimensional thymic environment. Nat. Immunol. 6, 143–151 (2005). 15654342 10.1038/ni1161

[37] M. A. Daniels, E. Teixeiro, J. Gill, B. Hausmann, D. Roubaty, K. Holmberg, G. Werlen, G. A. Holländer, N. R. J. Gascoigne, E. Palmer, Thymic selection threshold defined by compartmentalization of Ras/MAPK signalling. Nature 444, 724–729 (2006). 17086201 10.1038/nature05269

[38] S. Gras, L. Kjer-Nielsen, Z. Chen, J. Rossjohn, J. McCluskey, The structural bases of direct T-cell allorecognition: Implications for T-cell-mediated transplant rejection. Immunol. Cell Biol. 89, 388–395 (2011). 21301478 10.1038/icb.2010.150

[39] S. P. Foy, K. Jacoby, D. A. Bota, T. Hunter, Z. Pan, E. Stawiski, Y. Ma, W. Lu, S. Peng, C. L. Wang, B. Yuen, O. Dalmas, K. Heeringa, B. Sennino, A. Conroy, M. T. Bethune, I. Mende, W. White, M. Kukreja, S. Gunturu, E. Humphrey, A. Hussaini, D. An, A. J. Litterman, B. B. Quach, A. H. C. Ng, Y. Lu, C. Smith, K. M. Campbell, D. Anaya, L. Skrdlant, E. Y.-H. Huang, V. Mendoza, J. Mathur, L. Dengler, B. Purandare, R. Moot, M. C. Yi, R. Funke, A. Sibley, T. Stallings-Schmitt, D. Y. Oh, B. Chmielowski, M. Abedi, Y. Yuan, J. A. Sosman, S. M. Lee, A. J. Schoenfeld, D. Baltimore, J. R. Heath, A. Franzusoff, A. Ribas, A. V. Rao, S. J. Mandl, Non-viral precision T cell receptor replacement for personalized cell therapy. Nature 615, 687–696 (2023). 36356599 10.1038/s41586-022-05531-1 PMC9768791

[40] H. P. Arrol, L. D. Church, P. A. Bacon, S. P. Young, Intracellular calcium signalling patterns reflect the differentiation status of human T cells. Clin. Exp. Immunol. 153, 86–95 (2008). 18460013 10.1111/j.1365-2249.2008.03677.x PMC2432102

[41] S. Kircher, M. Merino-Wong, B. A. Niemeyer, D. Alansary, Profiling calcium signals of in vitro polarized human effector CD4^+^ T cells. Biochim. Biophys. Acta Mol. Cell Res. 1865, 932–943 (2018). 29626493 10.1016/j.bbamcr.2018.04.001

[42] S. This, D. Rogers, È. Mallet Gauthier, J. N. Mandl, H. J. Melichar, What’s self got to do with it: Sources of heterogeneity among naive T cells. Semin. Immunol. 65, 101702 (2023). 36463711 10.1016/j.smim.2022.101702

[43] K. E. Smith, A. O. Smith, A spectral enabled GAN for time series data generation. arXiv [cs.LG] (2021). http://arxiv.org/abs/2103.01904.

[44] Y. Zhang, Z. Wang, Z. Zhang, J. Liu, Y. Feng, L. Wee, A. Dekker, Q. Chen, A. Traverso, GAN-based one dimensional medical data augmentation. Soft Comput. 27, 10481–10491 (2023).

[45] M. M. Rahman, M. W. Rivolta, F. Badilini, R. Sassi, A systematic survey of data augmentation of ECG signals for AI applications. Sensors 23, 5237 (2023). 37299964 10.3390/s23115237 PMC10256074

[46] S. R. Achar, F. X. P. Bourassa, T. J. Rademaker, A. Lee, T. Kondo, E. Salazar-Cavazos, J. S. Davies, N. Taylor, P. François, G. Altan-Bonnet, Universal antigen encoding of T cell activation from high-dimensional cytokine dynamics. Science 376, 880–884 (2022). 35587980 10.1126/science.abl5311 PMC12302665

[47] H. Ide, T. Aoshi, M. Saito, W. V. Espulgar, J. C. Briones, M. Hosokawa, H. Matsunaga, K. Arikawa, H. Takeyama, S. Koyama, H. Takamatsu, E. Tamiya, Linking antigen specific T-cell dynamics in a microfluidic chip to single cell transcription patterns. Biochem. Biophys. Res. Commun. 657, 8–15 (2023). 36963175 10.1016/j.bbrc.2023.03.035

[48] J. F. Ashby, J. Schmidt, N. Kc, A. Kurum, C. Koch, A. Harari, L. Tang, S. H. Au, Microfluidic T Cell selection by cellular avidity. Adv. Healthc. Mater. 11, e2200169 (2022). 35657072 10.1002/adhm.202200169 PMC11468699

[49] M. A. Stockslager, J. S. Bagnall, V. C. Hecht, K. Hu, E. Aranda-Michel, K. Payer, R. J. Kimmerling, S. R. Manalis, Microfluidic platform for characterizing TCR-pMHC interactions. Biomicrofluidics 11, 064103 (2017). 29204244 10.1063/1.5002116 PMC5685811

[50] P. J. Attayek, S. A. Hunsucker, C. E. Sims, N. L. Allbritton, P. M. Armistead, Identification and isolation of antigen-specific cytotoxic T lymphocytes with an automated microraft sorting system. Integr. Biol. 8, 1208–1220 (2016). 10.1039/c6ib00168h PMC5138107 27853786

[51] L. Binan, J. Roy, S. Costantino, Opto-magnetic selection and isolation of single cells. Bio Protoc. 9, e3428 (2019). 10.21769/BioProtoc.3428 PMC7853932 33654925

[52] L. Binan, J. Mazzaferri, K. Choquet, L.-E. Lorenzo, Y. C. Wang, E. B. Affar, Y. De Koninck, J. Ragoussis, C. L. Kleinman, S. Costantino, Live single-cell laser tag. Nat. Commun. 7, 11636 (2016). 27198043 10.1038/ncomms11636 PMC4876456

[53] N. Desjardins-Lecavalier, G. Modica, S. Costantino, Laser-assisted single-cell labeling and capture. Methods Mol. Biol. 2614, 357–368 (2023). 36587135 10.1007/978-1-0716-2914-7_21

[54] N. Desjardins-Lecavalier, M. G. Annis, A. Nowakowski, A. Kiepas, L. Binan, J. Roy, G. Modica, S. Hébert, C. L. Kleinman, P. M. Siegel, S. Costantino, Migration speed of captured breast cancer subpopulations correlates with metastatic fitness. J. Cell Sci. 136, jcs260835 (2023). 37313743 10.1242/jcs.260835 PMC10657211

[55] K. H. Hu, J. P. Eichorst, C. S. McGinnis, D. M. Patterson, E. D. Chow, K. Kersten, S. C. Jameson, Z. J. Gartner, A. A. Rao, M. F. Krummel, ZipSeq: Barcoding for real-time mapping of single cell transcriptomes. Nat. Methods 17, 833–843 (2020). 32632238 10.1038/s41592-020-0880-2 PMC7891292

[56] M.-D. N. Pham, T.-N. Nguyen, L. S. Tran, Q.-T. B. Nguyen, T.-P. H. Nguyen, T. M. Q. Pham, H.-N. Nguyen, H. Giang, M.-D. Phan, V. Nguyen, epiTCR: A highly sensitive predictor for TCR-peptide binding. Bioinformatics 39, btad284 (2023). 37094220 10.1093/bioinformatics/btad284 PMC10159657

[57] L. Wang, X. Lan, Rapid screening of TCR-pMHC interactions by the YAMTAD system. Cell Discov. 8, 30 (2022). 35379810 10.1038/s41421-022-00386-2 PMC8979966

[58] P. Mombaerts, J. Iacomini, R. S. Johnson, K. Herrup, S. Tonegawa, V. E. Papaioannou, RAG-1-deficient mice have no mature B and T lymphocytes. Cell 68, 869–877 (1992). 1547488 10.1016/0092-8674(92)90030-g

[59] K. A. Hogquist, S. C. Jameson, W. R. Heath, J. L. Howard, M. J. Bevan, F. R. Carbone, T cell receptor antagonist peptides induce positive selection. Cell 76, 17–27 (1994). 8287475 10.1016/0092-8674(94)90169-4

[60] P. Mombaerts, A. R. Clarke, M. A. Rudnicki, J. Iacomini, S. Itohara, J. J. Lafaille, L. Wang, Y. Ichikawa, R. Jaenisch, M. L. Hooper, Mutations in T-cell antigen receptor genes alpha and beta block thymocyte development at different stages. Nature 360, 225–231 (1992). 1359428 10.1038/360225a0

[61] H. Pircher, K. Bürki, R. Lang, H. Hengartner, R. M. Zinkernagel, Tolerance induction in double specific T-cell receptor transgenic mice varies with antigen. Nature 342, 559–561 (1989). 2573841 10.1038/342559a0

[62] J. Roy, J. Mazzaferri, J. G. Filep, S. Costantino, A haptotaxis assay for neutrophils using optical patterning and a high-content approach. Sci. Rep. 7, 2869 (2017). 28588217 10.1038/s41598-017-02993-6 PMC5460230

[63] J. Mazzaferri, J. Roy, S. Lefrancois, S. Costantino, Adaptive settings for the nearest-neighbor particle tracking algorithm. Bioinformatics 31, 1279–1285 (2015). 25480371 10.1093/bioinformatics/btu793

[64] J. C. Crocker, D. G. Grier, Methods of digital video microscopy for colloidal studies. J. Colloid Interface Sci. 179, 298–310 (1996).

