# Supplementary material for: Machine learning predictions of T cell antigen specificity from intracellular calcium dynamics
Source: Sci Adv. 2024 Mar 6;10(10):eadk2298. doi: 10.1126/sciadv.adk2298 (PMC10917351; doi:10.1126/sciadv.adk2298)
Supplement: Supplementary file 1 — Figs. S1 to S9 Tables S1 to S3 [file sciadv.adk2298_sm.pdf]

Science Advances
AAAS

Supplementary Materials for

Machine learning predictions of T cell antigen specificity from intracellular calcium dynamics

Sébastien This *et al.*

Corresponding author: Santiago Costantino, santiago.costantino@umontreal.ca;
Heather J. Melichar, heather.melichar@mcgill.ca

Sci. Adv. **10**, eadk2298 (2024)
DOI: 10.1126/sciadv.adk2298

This PDF file includes:

Figs. S1 to S9
Tables S1 to S3

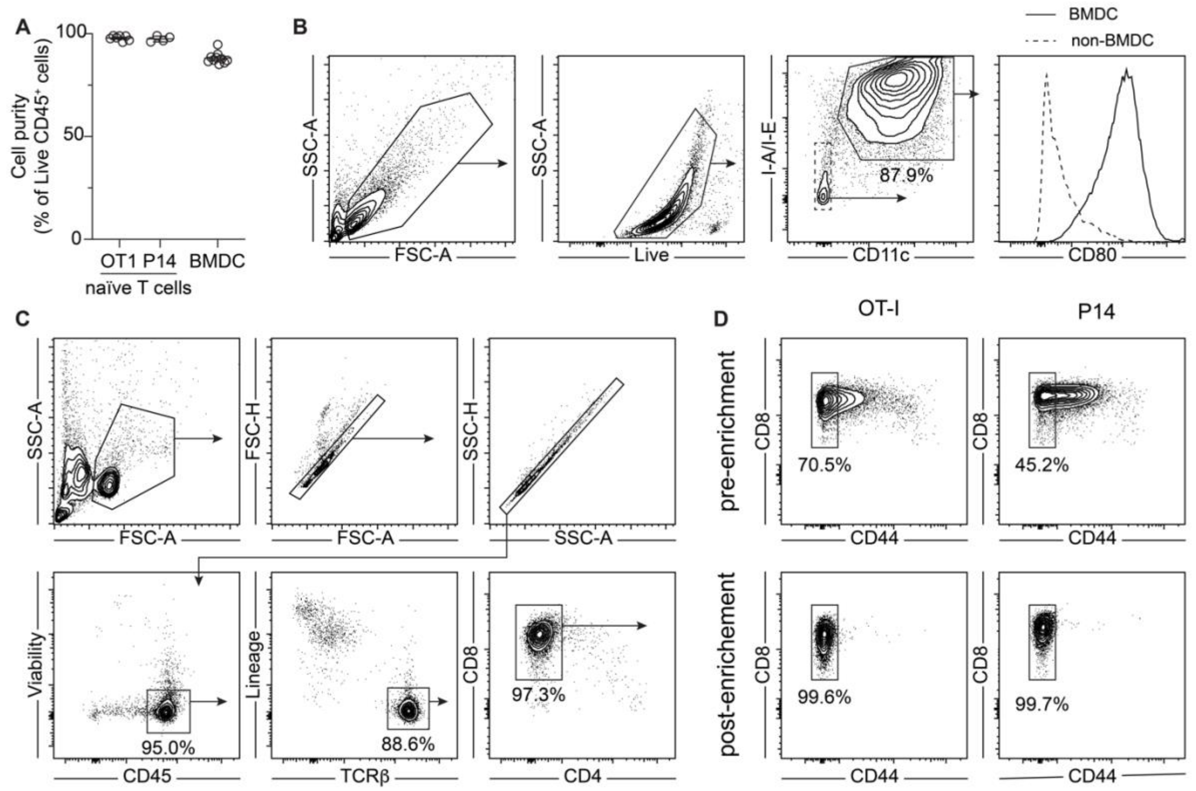

Fig. S1. Cell purities for the *in vitro* co-culture system (A) Purity of CD8$^+$ naïve T cells (Live Lin$^-$ CD45$^+$ TCRβ$^+$ CD8$^+$ CD44$^-$) and BMDC (Live CD11c$^+$ I-A/I-E$^+$) used in the co-culture assay as measured by flow cytometry. Error bars show SD. (n=7-11; Lin$^-$ = CD19$^-$ CD11c$^-$ NK1.1$^-$ CD11b$^-$ TCRγδ$^-$) **(B)** Gating strategy for the measure of BMDC purity and representative plot of CD80 expression on BMDC and non-BMDC as control. **(C & D)** Gating strategy to evaluate the purity of naïve CD8$^+$ OT-I and P14 T cells (Live Lin$^-$ CD45$^+$ TCRβ$^+$ CD8$^+$ CD44$^-$) as measured by flow cytometry. **(D)** Representative plots of naïve CD8$^+$ T cell frequency before and after magnetic enrichment of naïve OT-I and P14 T cells.

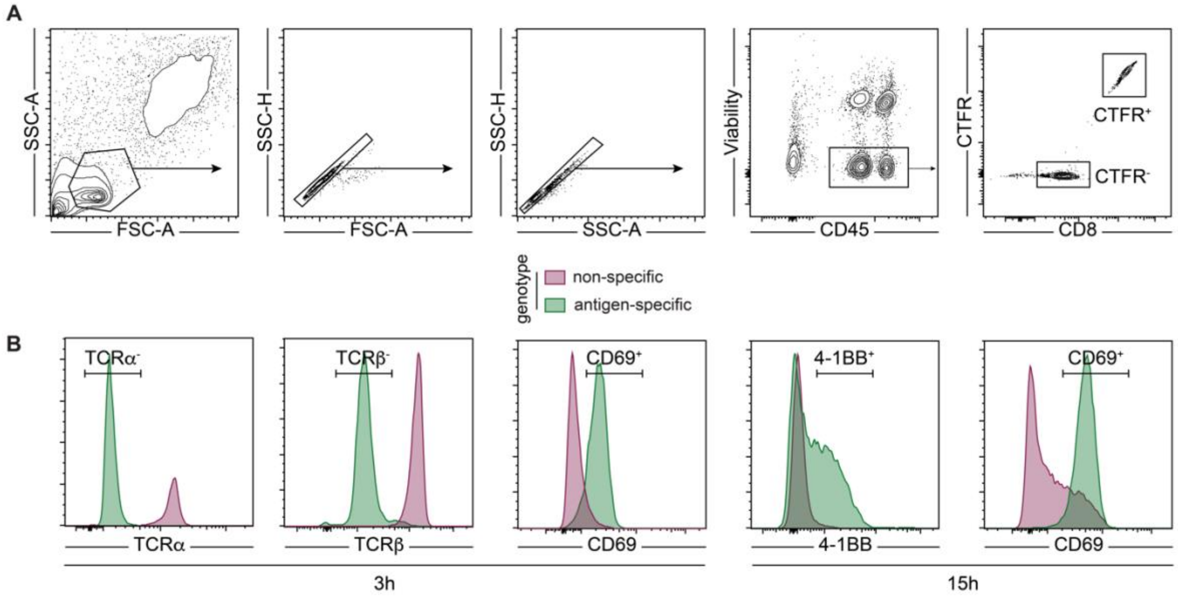

Fig. S2. Evaluation of monoclonal T cell activation by flow cytometry. (A) Gating strategy used for the identification of $CTFR^+$ and $CTFR^-$ $CD8^+$ T cells following co-culture. The genotype is determined based on which cells (OT-I or P14) were stained with CTFR prior to co-culture. **(B)** Representative distribution of TCRα, TCRβ, CD69 and 4-1BB expression on antigen-specific and non-specific T cells after 3 and 15 hours of co-culture as indicated.

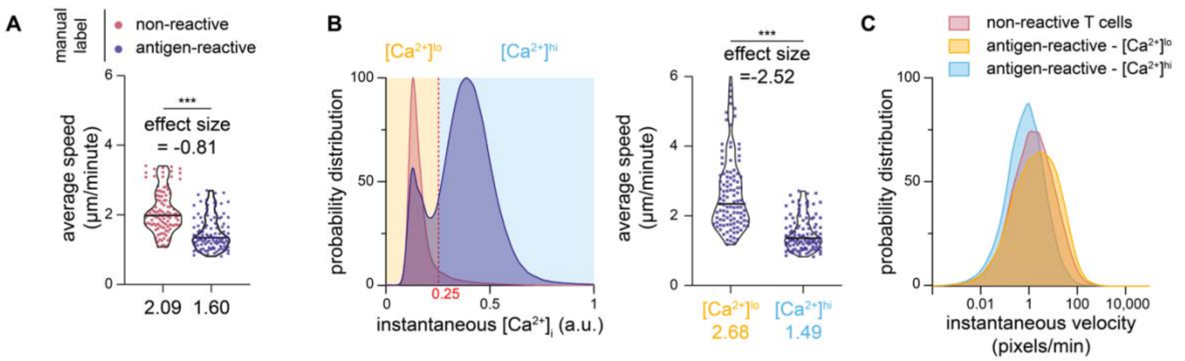

Fig. S3. Calcium signaling is associated with reduced T cell speed in the *in vitro* co-culture assay. (A) Quantification of average speed across the entire timelapse for all T cells based on manual labeling assignment. Horizontal lines in the violin plot show the median and numbers below show the average of the distribution. Effect size between both distributions is computed using the left condition as control. (n=111 fields of view over 8 independent experiments) **(B)** For each timelapse, an Otsu threshold (red dashed line; threshold=0.25) on the instantaneous Ca^{2+} concentration of all cells is used to separate $[Ca^{2+}]^{lo}$ from $[Ca^{2+}]^{hi}$ states. The average speed for all cells at timepoints where they are in either Ca^{2+} state is plotted on the right. **(C)** Distribution of instantaneous velocity in all cells for all timelapses according to antigen-specificity and Ca^{2+} concentration status. (n=111 fields of view; n=7173 antigen-reactive; n=7564 non-reactive T cells over 8 independent experiments; Mann-Whitney U-test). $[Ca^{2+}]_i$ = intracellular calcium concentration.

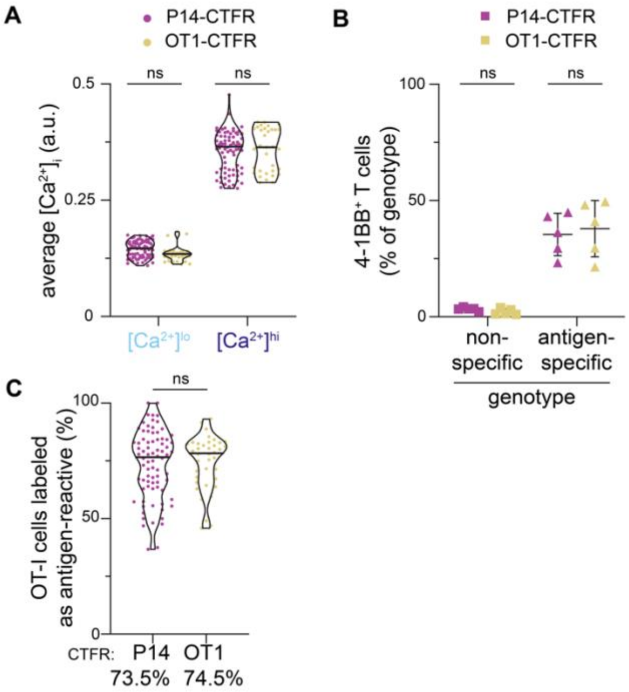

Fig. S4. CTFR staining of OT1 or P14 does not affect activation. (A) For each timelapse, two Gaussian distributions were fitted to the distribution of Ca^{2+} concentration across all cells (Fig. 1C). The mean of these distributions is used as a surrogate for the mean intracellular Ca^{2+} concentration of the $[Ca^{2+}]^{hi}$ and $[Ca^{2+}]^{lo}$ populations. **(B)** Flow cytometric measurement of 4-1BB expression after 15h co-culture. Error bars show SD. (n=5 culture wells over 8 independent experiments) **(C)** Frequency of OT-I, expressed as frequency of genotype, manually identified as antigen-reactive by four independent evaluators. Horizontal lines in the violin plots (A and C) show the median and numbers below show the average of the distribution. (n=78 P14-CTFR fields of view; n=38 OT1-CTFR fields of view over 8 independent experiments; Mann-Whitney U-test). $[Ca^{2+}]_i$ = intracellular calcium concentration.

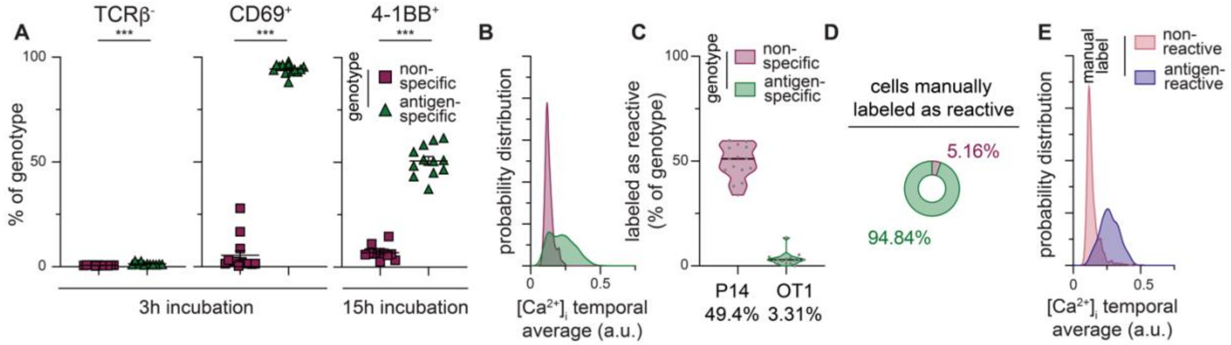

Fig. S5. Validation of the specificity of the co-culture model when using the gp33 peptide. **(A)** Flow cytometry assessment of surface TCRβ down-regulation, CD69 expression, and 4-1BB expression, 3h or 15h after co-culture initiation. Error bars indicate standard deviation (SD). (n=11-13 independent culture wells over 6 independent experiments; Mann-Whitney U-test) **(B)** Probability distribution of the average Ca^{2+} concentration over the entire timelapse according to antigen-specificity assignment. (n=1108 antigen-specific cells; n=1168 non-specific cells over 6 independent experiments) **(C)** Frequency of T cells, expressed as percentage of genotype, manually labeled as antigen-reactive. Horizontal lines in the violin plot show the median and numbers below show the average of the distribution. Individual fields of view are represented in gray. (n=15 fields of view over 6 independent experiments) **(D)** Detailed composition of cells manually labeled as antigen-reactive. Some of these are non-specific, making a false discovery rate for the manual labeling process of 5.16%. **(E)** Probability distribution of the average intracellular Ca^{2+} concentration according to manual assignment (n=594 cells labeled as antigen-reactive; n=1682 cells labeled as non-reactive over 6 independent experiments). $[Ca^{2+}]_i$ = intracellular calcium concentration.

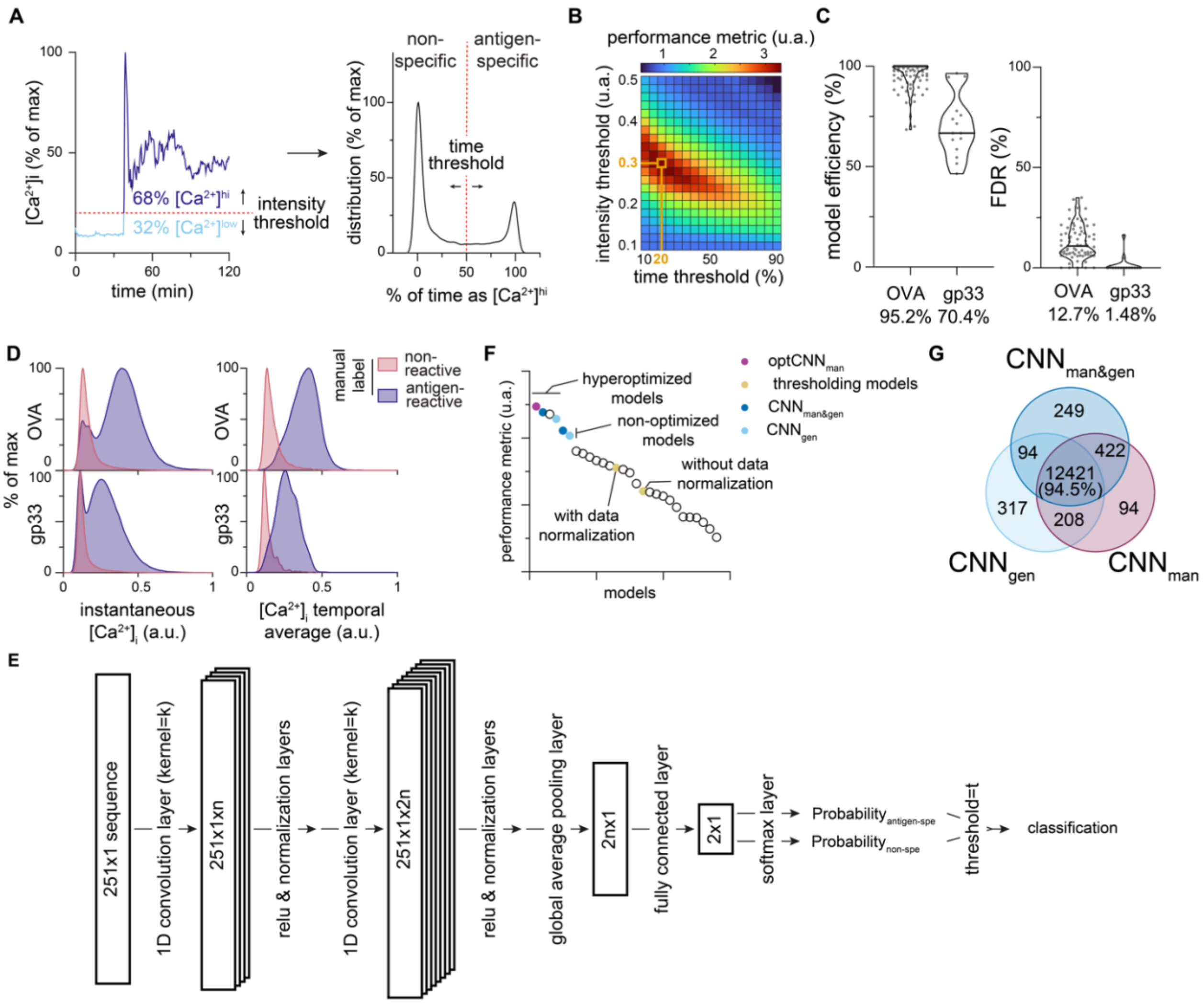

Fig. S6. Deep-learning approaches perform better than conventional methods for the classification of T cell activation based on Ca^{2+} fluctuations. (A-C) Use of conventional thresholding methods to classify T cell antigen-reactivity. **(A)** Varying both intensity and time thresholds (red lines) to predict antigen-specific from non-specific T cells - cells above the time threshold being classified as antigen-specific - we evaluate the performance (on the training dataset) for each pair of thresholds allows to pick an optimal pair of thresholds. **(B)** Performance of the prediction of antigen-specificity using each pair of thresholds during the training phase. The optimal pair (0.3, 20) of thresholds is highlighted with a thicker line. **(C)** Using these optimal thresholds, the model efficiency (frequency of cells labeled as antigen-reactive predicted as antigen-specific) and False Discovery Rate (FDR = frequency of cells mispredicted as antigen-specific) is computed on the evaluation dataset. Horizontal lines in the violin plot show the median and numbers below show the average of the distribution. Individual fields of view are

represented in gray. (n=73 OVA fields of view; n=15 gp33 fields of view over 9 OVA and 2 gp33 independent experiments) **(D)** Distribution of intracellular Ca^{2+} concentration for all timepoints (left) or the average Ca^{2+} concentration over the entire timelapse (right) according to manual labelling assignment and the peptide used in the co-culture. Each distribution is normalized to its mode. (OVA: n=4584 antigen-reactive cells, n=4864 non-reactive cells; gp33: n=1108 antigen-reactive cells, n=1168 non-reactive cells over 9 OVA and 2 gp33 independent experiments). **(E)** Detailed structure of $optCNN_{man}$. Parameters (n, k & t) are indicated in Table S2. **(F)** Performance metrics of all models shown in Table S2 and ranked according to their performance. A few selected models are highlighted. **(G)** Venn diagram showing the overlap between predictions of the 3 hyperoptimized models: CNN_{man}, CNN_{gen} & $CNN_{man\&gen}$, the subscript referring to the ground truth used for training (see Table S2). $[Ca^{2+}]_i$ = intracellular calcium concentration.

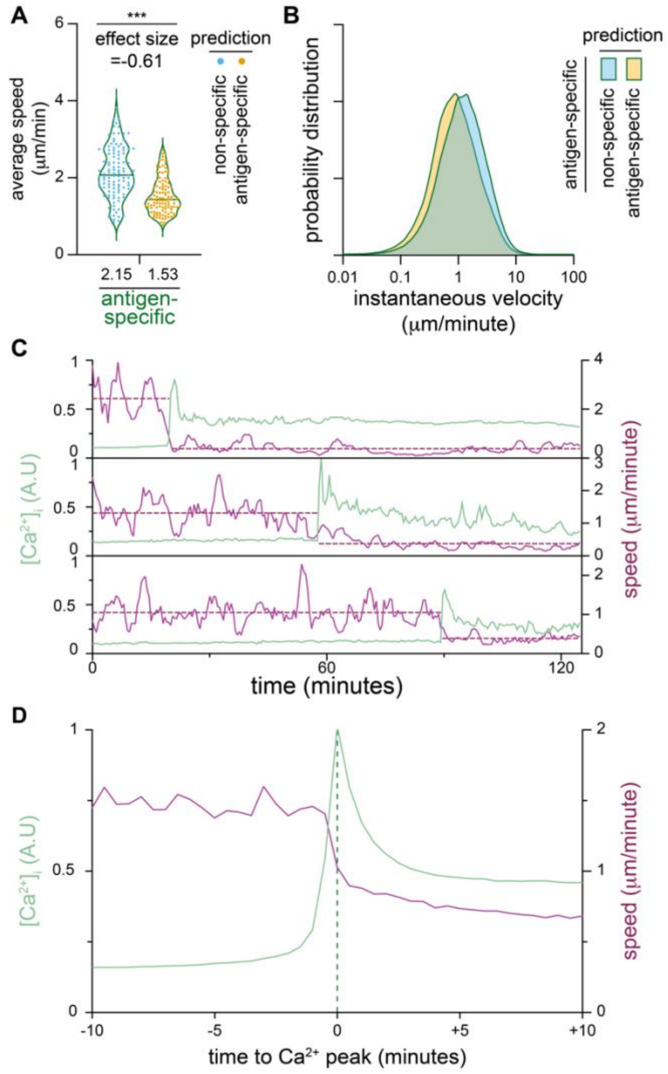

Fig. S7. Intracellular Ca^{2+} dynamics of cells predicted as activated negatively correlates with their motility. (A-D) T cell motility following coculture with OVA-presenting BMDC. **(A)** Average speed across the entire timelapse for all T cells based on $optCNN_{man}$ prediction. Every dot represents the average speed of all cells in each field of view. Horizontal lines in the violin plot show the median and numbers below show the average of the distribution. Effect size between both distributions is computed using the left condition as control. (n=111 fields of view over 8 independent experiments) **(B)** Distribution of instantaneous velocity in all antigen-specific T cells for all timelapses according to $optCNN_{man}$ prediction. (n=5947 predicted as antigen-specific and n=8789 predicted as non-specific T cells over 8 independent experiments) **(C)** Representative dynamics of intracellular Ca^{2+} concentration and instantaneous speed across the entire timelapse for 3 selected antigen-specific T cells. The green line represents the intracellular Ca^{2+}

concentration dynamics. The magenta line represents the distance travelled by the cell between two timeframes (instantaneous speed), smoothed out (average) over a 10 frames window for readability. The magenta dotted line represents the average speed before and after the Ca^{2+} peak. **(D)** Average dynamics of intracellular Ca^{2+} concentration and instantaneous speed before and after initial antigen encounter. Cells predicted as activated and exhibiting a initial spike in Ca^{2+} levels within the imaging window where identified and aligned so that the first peak of increased Ca^{2+} is synchronized for all cells. The intracellular Ca^{2+} concentration and the velocity 10 frames before and after the peak is averaged for all relevant T cells. (n=2443 cells over 8 independent experiments). $[Ca^{2+}]_i$ = intracellular calcium concentration.

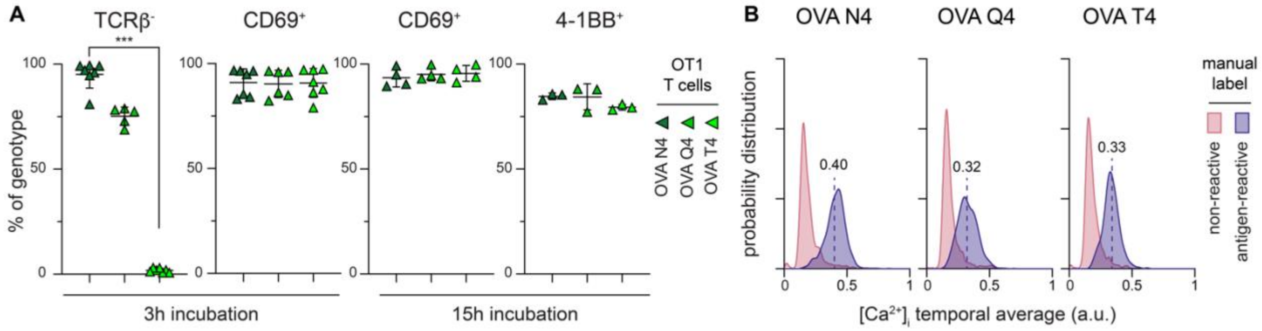

Fig. S8. OT1 T cells mount robust responses to altered peptide ligand stimulation. (A) Flow cytometry assessment of surface TCRβ down-regulation, CD69 expression, and 4-1BB expression, 3h or 15h after co-culture initiation. Error bars indicate standard deviation (SD). (n=3-6 independent wells over 5 independent experiments; ***=p<0.005 – Kruskal-Wallis one-way ANOVA with Dunn's multiple comparison *post-hoc* test) **(B)** Probability distribution of the average Ca^{2+} concentration over the whole timelapse according to manually assigned antigen-reactivity. (OVA N4: n=1184 antigen-reactive cells; n=1051 non-reactive cells. OVA Q4: n=972 antigen-reactive cells; n=1161 non-reactive cells. OVA T4: n=1155 antigen-reactive cells; n=1089 non-reactive cells over 5 independent experiments). Dotted line and number represent the mean of intracellular Ca^{2+} concentration for the antigen-reactive T cell population. $[Ca^{2+}]_i$ = intracellular calcium concentration.

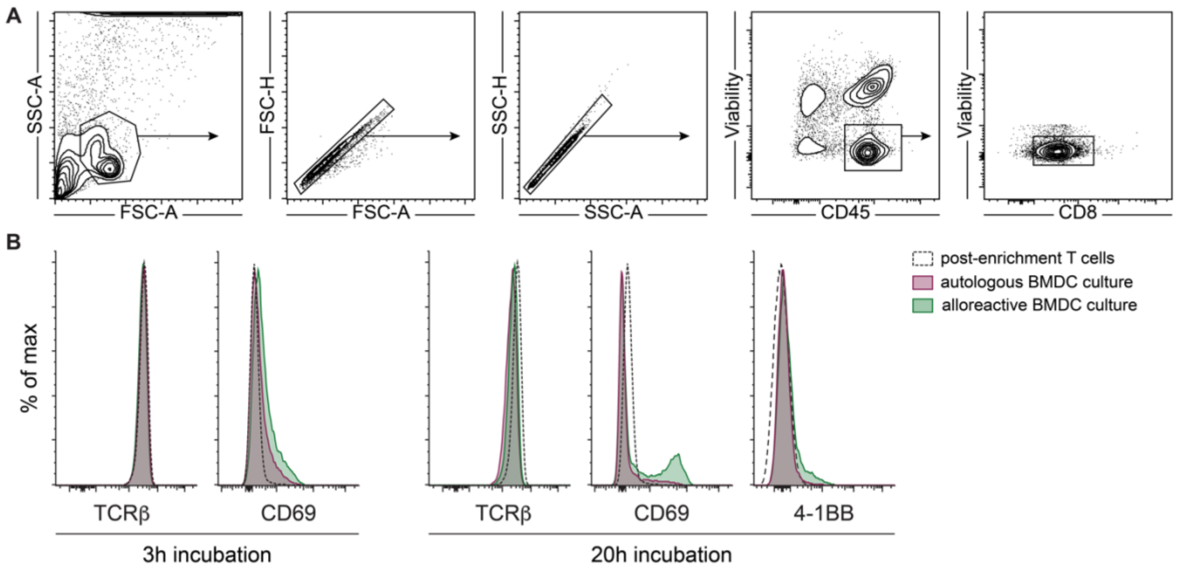

Fig. S9. Evaluation of polyclonal T cell activation by flow cytometry. (A) Gating strategy used for the identification of $CD8^{+}$ T cells following MLR co-culture. **(B)** Representative distribution of TCRβ, CD69 and 4-1BB expression by T cells in autologous and alloreactive culture conditions, after 3 and 20 hours of co-culture as indicated.

	timelapses			# cells	
	peptide	CTFR	#	genotype	manual label
training dataset	OVA	P14	29	3513 antigen-specific<br>6252 non-specific	2673 antigen-reactive<br>7096 non- reactive
		OT-I	27		
	no peptide	P14	6		
		OT-I	6		
evaluation dataset	OVA	P14	52	4584 antigen-specific<br>4864 non-specific	3380 antigen- reactive<br>6068 non- reactive
		OT-I	21		
	gp33	P14	6	1108 antigen-specific<br>1168 non-specific	594 antigen- reactive<br>1682 non- reactive
		OT-1	9		

Table S1. Distribution of all timelapses among training and evaluation datasets. The table highlights the peptide, which cells have been stained with CTFR, and the number of positive and negative cells in each group.

	ground truth	hyperoptimization	data normalization	use of derivative	use of speed	data augmentation	efficiency (OVA)	FDR (OVA)	efficiency (gp33)	FDR (gp33)	performance metric (u.a.)	ranking
**exploration of optimal ML architecture**												
clustering approaches												
k-means clustering	gen						49.8 ± 1.87	9.78 ± 1.77	45.0 ± 4.00	3.32 ± 2.67	1.283	24
KNN classification	gen						84.4 ± 1.33	6.25 ± 0.79	42.0 ± 4.94	0.75 ± 0.56	1.620	22
SVM based approaches												
SVM classifier	gen						84.4 ± 1.43	8.80 ± 0.91	44.7 ± 4.85	0.74 ± 0.42	1.645	20
SVM classifier	gen	Y					84.7 ± 1.37	8.94 ± 0.91	44.2 ± 4.75	0.68 ± 0.42	1.632	21
SVM classifier	gen	Y	Y				85.1 ± 1.31	8.81 ± 0.95	71.3 ± 2.96	0.91 ± 0.55	2.639	14
recursive SVM classifier	gen	Y	Y				78.6 ± 1.65	7.02 ± 0.81	61.6 ± 3.39	0.59 ± 0.34	2.142	18
recursive SVM classifier	gen	Y	Y	Y			74.8 ± 1.90	5.21 ± 0.94	67.2 ± 3.14	0.92 ± 0.60	2.323	16
recursive SVM classifier	man	Y	Y	Y			56.2 ± 2.60	3.28 ± 0.70	49.0 ± 3.53	0.12 ± 0.12	1.480	23
ensemble approaches												
tree classifier	man	Y	Y	Y			87.1 ± 1.47	12.6 ± 1.27	91.3 ± 1.62	18.1 ± 2.09	1.935	19
KNN classifier	man	Y	Y	Y			31.6 ± 2.50	9.11 ± 2.94	35.0 ± 1.93	2.96 ± 1.48	1.033	25
discriminant classifier	man	Y	Y	Y			87.9 ± 1.24	9.82 ± 1.20	87.8 ± 2.17	14.0 ± 1.88	2.383	15
deep learning approaches												
CNN	man		Y				95.2 ± 0.58	7.88 ± 0.85	90.4 ± 1.58	8.27 ± 1.77	3.610	7
1D-Alexnet	man		Y				77.1 ± 1.89	4.92 ± 1.00	63.9 ± 3.63	0.92 ± 0.64	2.266	17
LSTM	man		Y				88.9 ± 1.24	5.60 ± 0.84	71.9 ± 3.59	0.17 ± 0.12	3.059	12
CNN-LSTM	man		Y				85.2 ± 1.62	5.72 ± 1.00	72.6 ± 2.99	0.52 ± 0.43	2.964	13
FC-NN	man		Y				88.8 ± 1.27	8.82 ± 0.96	79.5 ± 2.57	1.32 ± 0.55	3.258	10
**exploration of optimal training paramteres**												
ground truth exploration												
CNN	man		Y				95.2 ± 0.58	7.88 ± 0.85	90.4 ± 1.58	8.27 ± 1.77	3.610	7
CNN	gen		Y				91.8 ± 1.08	6.60 ± 0.78	77.8 ± 2.49	2.00 ± 0.66	3.539	8
recursive CNN	gen		Y				90.9 ± 1.11	5.03 ± 0.71	74.8 ± 2.93	1.29 ± 0.65	3.430	9
CNN	gen		Y	Y			94.3 ± 0.76	6.27 ± 0.71	81.1 ± 2.68	1.85 ± 0.77	4.072	6
CNN	gen		Y		Y		91.4 ± 1.07	8.35 ± 0.78	77.0 ± 2.78	2.82 ± 0.71	3.175	11
CNN	man&gen		Y				91.5 ± 1.07	4.82 ± 0.74	81.0 ± 2.48	1.57 ± 0.91	4.226	5
hyperoptimization												
**CNN**	**man**	**Y**	**Y**				**93.7 ± 0.83**	**6.26 ± 0.82**	**88.6 ± 1.97**	**2.90 ± 1.34**	**4.944**	**1**
CNN	gen	Y	Y				95.1 ± 0.72	6.75 ± 0.75	86.2 ± 1.85	2.64 ± 0.76	4.577	4
CNN	man&gen	Y	Y				91.4 ± 1.09	5.73 ± 0.84	87.2 ± 1.57	2.72 ± 1.50	4.773	2
data augmentation												
CNN	man	Y	Y			Y	93.1 ± 0.90	6.04 ± 0.80	86.3 ± 2.17	2.52 ± 1.02	4.717	3

Table S2. Training method, performance, and ranking based on the weighted performance metric of selected ML models. First, we explore amongst well known ML architectures the best performing model with the dataset. We use the term recursive to describe the process of training a first model on Ca^{2+} fluctuation, the prediction of which is used as ground-truth for a second SVM model. Data shows average ± SEM. 'man' = manual label used as ground-truth; 'gen'= genotype used as ground-truth; 'man&gen' = antigen-specific T cells manually labeled as antigen-reactive used as ground-truth; KNN = k-nearest neighbors, SVM = Support Vector Machine, LSTM = Long Short-Term Memory networks, FC-NN = fully connected neural networks, CNN = Convolutional Neural Network).

ML structure	man	gen	man&gen
number of neurons (n)	30	30	60
kernel size (k)	8	8	2
number of classes	2	2	3
**training parameters**			
optimizer	adam	adam	adam
minibatch size	100	25	50
internal validation	15%	15%	15%
**prediction**			
threshold (t)	0.46	0.5	0.4

Table S3. Parameters used for the generation, the training, and the prediction of antigen-specificity with the three hyperoptimized models. Letters in parentheses reflect parameters presented in Fig. S6
